# Fluorescent Indicators For Biological Imaging of Monatomic Ions

**DOI:** 10.3389/fcell.2022.885440

**Published:** 2022-04-27

**Authors:** Sheng-Yi Wu, Yi Shen, Irene Shkolnikov, Robert E. Campbell

**Affiliations:** ^1^ Department of Chemistry, University of Alberta, Edmonton, AB, Canada; ^2^ Department of Medical Sciences, University of Victoria, Victoria, BC, Canada; ^3^ Department of Chemistry, The University of Tokyo, Tokyo, Japan

**Keywords:** monatomic ions, small molecule-based indicators, genetically encoded indicators, fluorescence imaging, protein engineering

## Abstract

Monatomic ions play critical biological roles including maintaining the cellular osmotic pressure, transmitting signals, and catalyzing redox reactions as cofactors in enzymes. The ability to visualize monatomic ion concentration, and dynamic changes in the concentration, is essential to understanding their many biological functions. A growing number of genetically encodable and synthetic indicators enable the visualization and detection of monatomic ions in biological systems. With this review, we aim to provide a survey of the current landscape of reported indicators. We hope this review will be a useful guide to researchers who are interested in using indicators for biological applications and to tool developers seeking opportunities to create new and improved indicators.

## 1 Introduction

The interactions between biomolecules and inorganic ions are central to the chemistry of life. The most abundant monatomic inorganic ions in biology systems include sodium (Na^+^), potassium (K^+^), calcium (Ca^2+^), iron (Fe^2+^/Fe^3+^) and chloride (Cl^−^). Trace ions include zinc (Zn^2+^), molybdenum (Mo^+^), cobalt (Co^+^), copper (Cu^2+^/Cu^+^), and manganese (Mn^2+^) (Frieden, 1972). Together, these ions play important roles in physiology including serving as electrolytes, maintaining osmotic pressure and pH, and enabling neuronal activities. Likewise, some ions are incorporated into biologically relevant molecules such as cofactors that facilitate enzyme catalysis (Ainscough and Brodie, 1976). Important questions about the molecular basis of life rest on our understanding of ion dynamics in biological systems. Research into these questions relies on a diverse and highly optimized molecular toolkit for measuring ion concentration, mapping ion localization, and tracking ion flux with high spatiotemporal resolution.

There are a variety of non-imaging methods for measuring concentrations of metal ions. These methods include flame or graphite furnace atomic absorption spectroscopy (AAS) and inductively coupled plasma mass spectrometry (ICP-MS) (Ammann, 2007; Pröfrock and Prange, 2012; Cerchiaro et al., 2013). AAS analyzes only one element at a time, while the ICP-MS can quantitatively measure multiple elements simultaneously. While these methods can detect metal ions with very high sensitivity (parts per billion for AAS and parts per trillion for ICP-MS), they cannot distinguish between bound and free ions, or detect dynamic changes in ion concentrations with spatiotemporal resolution. Free ion concentration is of significant relevance to biologists because bound ions cannot be translocated across organelle or plasma membrane through ion channels or transporters. The adaptation of mass spectrometry with fixed biological samples has enabled elemental mapping at a cellular or subcellular level (Qin Z. et al., 2011). However, these techniques cannot provide dynamic information because they are destructive and are not suitable for use with living tissues. Ion-specific electrodes do provide real-time measurements at a single-cell level, but this technique is challenging and often impractical for measuring multiple cells simultaneously or at a subcellular level.

Imaging technology provides the spatiotemporal information of ion dynamics that eludes the aforementioned technologies. Dynamic changes of ion concentrations are the foundation of many important intra- and intercellular processes and reflect cellular responses to environmental perturbations (Leybaert and Sanderson, 2012; Yurinskaya et al., 2020). Measuring these ion concentrations enables inquiry into ion regulation and transportation, therefore tools for such measurements in tissue and whole organisms are of particular interest. Ion-sensitive indicators, coupled with light microscopy, are indispensable tools for the direct visualization of ion dynamics in multiple cells simultaneously with subcellular resolution.

Two categories of the fluorescent indicators are commonly used: small molecule-based synthetic indicators and genetically encodable protein-based indicators. Several properties are important for comparing the indicators: 1) brightness, the product of extinction coefficient (EC, the ability to absorb photons) and quantum yield (QY, the probability to emit a photon with every photon absorbed); 2) sensitivity, the amplitude of an indicator response to the ligand concentration in the detectable range, which is typically quantified as the maximum fluorescence change ∆*F*/*F*_0_ or ratio change ∆*R*/*R*_0_; 3) affinity, which is usually quantified by the dissociation constant (*K*_d_); 4) specificity, the ability of the indicator to recognize the intended target rather than unintended targets; 5) photostability, the ability of a fluorophore to resist photobleaching; 6) pH sensitivity, which is characterized by the apparent p*K*_a_ (the acid dissociation constant), the pH at which the fluorescence is half of its maximum value; 7) kinetics, the rate at which the indicator responds to the ligand concentration change and is measured by the association rate constant *k*_on_ and the dissociation rate constant *k*_off_; and finally, 8) targetability, the potential to be expressed in a specific cell type or a subcellular compartment.

Small molecule-based indicators and genetically encoded indicators are both associated with general advantages and disadvantages. Small molecule-based indicators are typically brighter and more photostable, but their use can be complicated by loading procedures and difficulties in subcellular or cell-specific targeting (Russell, 2011). Genetically encodable indicators can typically be expressed by the cellular machinery and trafficked to a specific location of the cell. However, applications can be limited by their relatively lower brightness and photostability. In this review, we aim to provide a detailed overview of the various types of indicators for monatomic ions, and compare them with respect to the properties listed above.

## 2 Ca^2+^ Indicators

### 2.1 The Role of Ca^2+^ in Cell Physiology

Ca^2+^ is involved in many central physiological activities (Clapham, 2007; Brini et al., 2014). As a universal second messenger, Ca^2+^ regulates cellular activities including cytoskeletal motility, phosphorylation and dephosphorylation-dependent enzymatic activity, and secretion of biomolecules such as neurotransmitters. During propagation of an action potential, voltage-sensitive Ca^2+^ channels on neurons open to allow an influx of Ca^2+^ (Brini et al., 2014). At a synapse, this influx of Ca^2+^ ions leads to neurotransmitter release and signal propagation. Because Ca^2+^ undergoes dramatic translocations during signaling events, being able to measure Ca^2+^ concentration dynamics is of particular importance.

The local concentration of Ca^2+^ varies from nanomolar level to millimolar level in cells. In the resting state, the intracellular concentration is typically maintained at approximately 100 nM in neurons (Khodorov et al., 1993; Bagur and Hajnóczky, 2017). Extracellular Ca^2+^ is typically maintained at 1 mM, 10,000-fold greater than the resting intracellular concentration. Ca^2+^ indicators with different affinities are developed to accommodate the wide span of Ca^2+^ concentrations in various biological contexts.

### 2.2 Small molecule-based Ca^2+^ indicators

**FIGURE 1:**
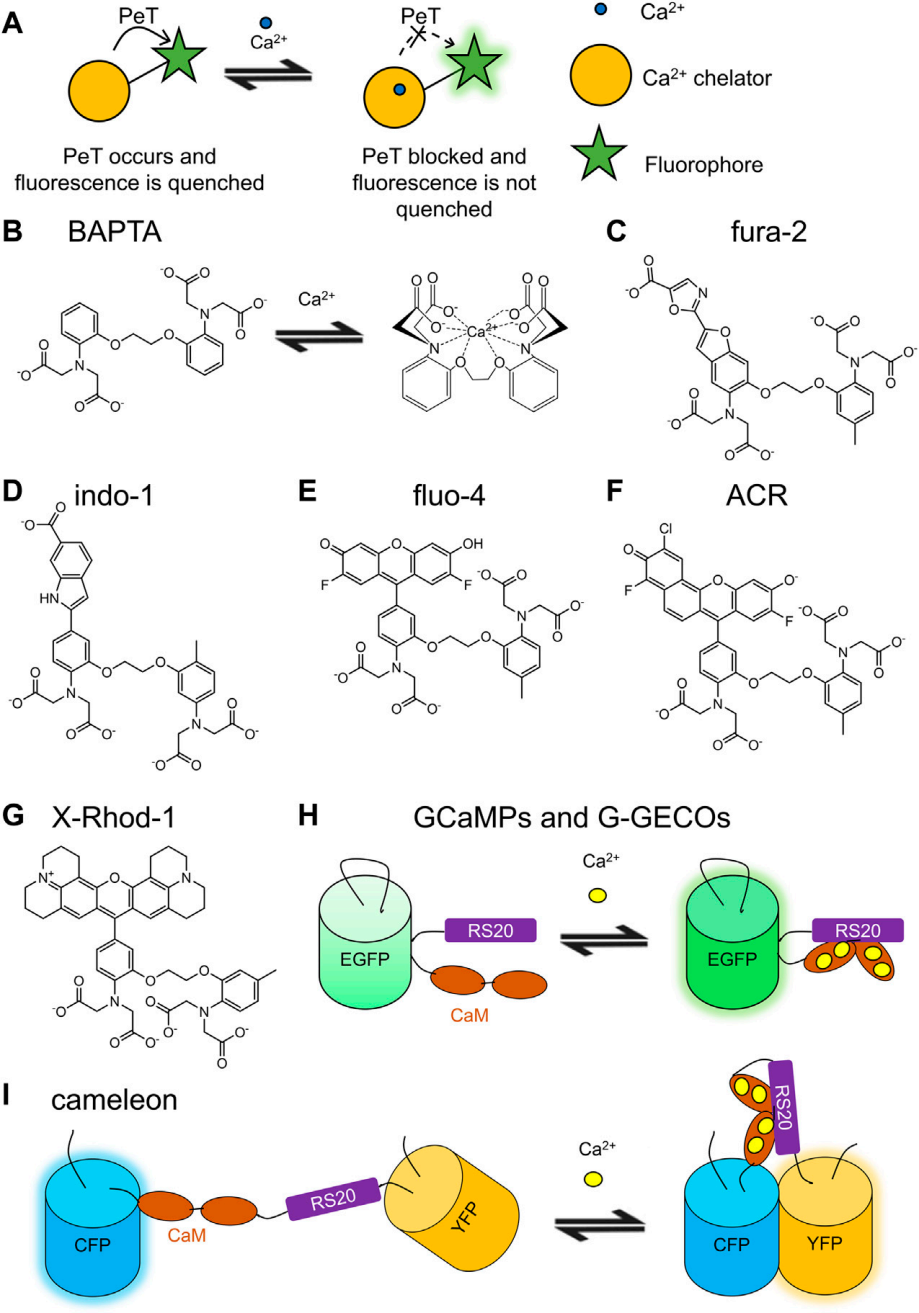
Schematic representations and molecular structures of selected small molecule-based Ca^2+^ indicators and schematic representations of selected GECIs. **(A)** Photoinduced electron transfer (PeT) is the typical mechanism used by small molecule-based Ca^2+^ indicators (Aigner et al., 2011, 2). For indicators with positive responses, the fluorophore is quenched by the electron-rich Ca^2+^ chelator *via* PeT in the unbound state. The binding of Ca^2+^ reduces PeT-induced quenching, leading to brighter fluorophore. **(B)** BAPTA is the Ca^2+^-binding motif for many small molecule-based Ca^2+^ indicators (Tsien, 1980). The binding of Ca^2+^ constrains the molecular structure of BAPTA, leading to a spectral change. **(C)** Fura-2 and **(D)** indo-1 are both derived from BAPTA and exhibit blue to green emission (Grynkiewicz et al., 1985). **(E)** Fluo-4 is based on a modified fluorescein fluorophore and exhibits bright green emission (Gee et al., 2000). **(F)** Rhodamine-based Asante Calcium Red (ACR) and **(G)** X-Rhod-1 exhibit red fluorescence (Tsien and Minta, 1991; Hyrc et al., 2013). **(H)** GCaMPs and G-GECOs are based on CaM/RS20 and cpEGFP (Nakai et al., 2001; Zhao et al., 2011). **(I)** Cameleon series is FRET-based GECIs, which show increased YFP fluorescence with Ca^2+^ binding (Miyawaki et al., 1997).

Small molecule-based Ca^2+^ indicators (Figure 1) typically contain a fluorophore and a Ca^2+^ chelator and often exhibit an increase in fluorescence in response to Ca^2+^. Ca^2+^ indicators are typically designed to operate through a photo-induced electron transfer (PeT) mechanism—a process in which an electron is transferred from the excited state electron donor, typically the Ca^2+^ chelator, to the electron acceptor, typically the fluorophore (Figure 1A) (Aigner et al., 2011). In absence of Ca^2+^, the electron-rich Ca^2+^ chelator quenches the fluorophore *via* PeT as the electrons from the chelator are transferred to the low-energy molecular orbital of the fluorophore and prevent photo-excited electrons in the high-energy molecular orbital from returning to the low-energy molecule orbital. In presence of Ca^2+^, this PeT mechanism is interrupted, allowing photo-excited electrons in the high-energy molecular orbital to return to the unoccupied low-energy molecular orbital by emitting fluorescence (Carter et al., 2014). The fluorescence of PeT-dependent small molecule-based Ca^2+^ indicators can be modulated by the presence of electron-rich or electron-withdrawing groups nearby.

#### 2.2.1 BAPTA and BAPTA-Based Ca^2+^ Indicators

The development of BAPTA [1, 2-bis (*o*-aminophenoxy) ethane-*N*, *N*, *N*′, *N*′-tetraacetic acid], an EGTA [ethylene glycol-bis (β-aminoethyl ether)-*N*, *N*, *N*′, *N*′-tetraacetic acid] analogue, in 1980, was a milestone in the history of small molecule-based fluorescent Ca^2+^ indicator development (Tsien, 1980). BAPTA coordinates Ca^2+^ through the four carbonyl oxygens, two ether oxygens, and the nitrogen of tertiary amine groups (Figure 1B). BAPTA binds Ca^2+^ with a *K*_d_ of 1.1 *×* 10^−7^ M, an affinity 10^5^ fold greater than that for Mg^2+^. Structurally, BAPTA differs from EGTA by replacing the methylenes between the nitrogen and the ether oxygen with a benzene ring, which renders the molecule fluorescent. Upon Ca^2+^ binding, the nitrogen atoms are twisted away from the same plane of the nearby benzene ring due to the structural constraint. This results in a major shift in the absorbance spectrum. The absorbance peaks at 254 nm (EC = 1.6 × 10^4^ M^−1^cm^−1^) and 287 nm (EC = 5.6 *×* 10^3^ M^−1^cm^−1^) in absence of Ca^2+^ and at 274 nm (EC = 4.2 *×* 10^3^ M^−1^cm^−1^) and 203 nm (EC = 4.1 *×* 10^4^ M^−1^cm^−1^) with 1 mM Ca^2+^.

Derivatives of BAPTA were explored with the goal of creating improved Ca^2+^ indicators. Quin-2 is a green fluorescent indicator derived from BAPTA that exhibits a positive response upon Ca^2+^ binding (Tsien and Pozzan, 1989). Its maximum excitation wavelength is at 339 nm and its maximum emission wavelength is at 492 nm. With a *K*_d_ of 115 nM for Ca^2+^, quin-2 is suitable for cytosolic Ca^2+^ detection. Its *K*_d_ for Mg^2+^ is in millimolar range, making quin-2 specific for Ca^2+^. Quin-2 has a EC < 5000 M^−1^cm^−1^ and QY of 0.03–0.14. Indo-1 (Figure 1D) and fura-2 (Figure 1C) are two bright and ratiometric Ca^2+^ indicators (Grynkiewicz et al., 1985). Both indicators have a *K*_d_ around 250 nM, slightly higher than that of quin-2. Indo-1 emits cyan light at 485 nm in a Ca^2+^ free environment and violet light at 410 nm in a Ca^2+^ rich environment, with excitation peaks at 349 and 331 nm, respectively. The ratiometric change in emission spectra upon Ca^2+^ binding allows emission ratiometric measurement. In contrast, fura-2 has a minor shift in emission peak from 512 to 505 nm with an increasing Ca^2+^ concentration. The excitation peak undergoes a larger blue shift from 362 to 335 nm, providing an opportunity for excitation ratiometric measurement. The QY increases from 0.23 to 0.49. Fura-2 enables applications using fluorescence microscopy with a single or dual-excitation setup. Fura-2 has a low affinity for Mg^2+^ (*K*_d_ = 1–2 mM) that allows intracellular Ca^2+^ imaging in various animal cells (Malgaroli et al., 1987).

BAPTA and BAPTA-based indicators were made cell-permeable by synthetic modification with acetoxymethyl (AM) ester groups to mask their carboxylate groups. These AM esters are readily hydrolyzed by cytosolic esterase as was originally demonstrated with human erythrocytes and rat mast cells (Tsien, 1981, 1983). This method is also used for other indicators with carboxylate groups including the ones for, Zn^2+^, K^+^, Mg^2+^, Na^+^, and H^+^.

#### 2.2.2 Fluorescein-Based Ca^2+^ Indicators

Fluorescein was used as the template to make various cell-compatible green indicators including fluo-1, fluo-2, fluo-3, fluo-4 (Figure 1E), fluo-8 series, Oregon green BAPTA-1 (OGB1), and Cal-520 (Minta et al., 1989; Gee et al., 2000; Lock et al., 2015). These green fluorescent indicators generally have higher sensitivity and faster kinetics than those of the BAPTA-based indicators mentioned above and bind Ca^2+^ with various affinities that allow detection in different cellular environments (Lock et al., 2015). For example, Cal-520 responds to Ca^2+^ faster than fluo-8H and provides a robust signal with a low signal-to-noise ratio (SNR) in subcellular regions of neurons. The fluo-8 series contains indicators with affinities ranging from 232 nM to 1.8 μM.

#### 2.2.3 Rhodamine-Based Ca^2+^ Indicators

Red-shifted Ca^2+^ indicators provide unique advantages for live-cell imaging: red light penetrates deeper into tissues and allows simultaneous imaging with green indicators. The rhodamine-based rhod2 is maximally excited by light at 553 nm and maximally emits light at 576 nm (Minta et al., 1989). The QY ranges from 0.03 (Ca^2+^ free) to 0.10 (excess of Ca^2+^) and the *K*_d_ value is 0.52 μM. Red-shifted and more sensitive variants such as rhod4 (Miranda et al., 2012; Lock et al., 2015)), rhod5N (Soibinet et al., 2008), Asante Calcium Red (ACR) (Figure 1F) (Hyrc et al., 2013), and X-Rhod-1 (Figure 1G) (Tsien and Minta, 1991) were further developed (Lock et al., 2015). Rhod5N has a maximum 150-fold change. ACR is excited at 537 nm and emits photons at 654 nm (Hyrc et al., 2013).

### 2.3 Genetically Encodable Ca^2+^ Indicators

Genetically encodable Ca^2+^ indicators (GECIs) share a general design that consists of a binding domain and a reporter domain. The reporter domain is typically a fluorescent protein (FP). Common choices for binding domains are the calmodulin (CaM)/CaM-binding peptide pair and troponin-C. Ca^2+^-dependent conformational changes of the binding domain cause changes of the FP chromophore environment, which in turn alters the fluorescence profile, allowing the monitoring of the Ca^2+^ concentration dynamics. A large collection of GECIs is available with various colours and affinities.

#### 2.3.1 FRET-Based Ca^2+^ Indicators

The development of fluorescent indicators has benefited from the expansion of the palette of FPs that have been discovered in nature or engineered from natural FPs. The currently available FP variants span the entire visible spectrum. A pair of FPs with different colours flanking at the ends of the Ca^2+^-binding domain enables the development of Ca^2+^ indicators based on Förster resonance energy transfer (FRET) — a phenomenon where the excited donor chromophore of the FP pair transfers its energy directly to the acceptor chromophore without photons emitted (Broussard and Green, 2017). FRET efficiency is inversely proportional to the 6th power of the distance between two chromophores (Förster, 1948) and Ca^2+^ binding induced conformational change typically reduces the distance, leading to an increase in FRET efficiency, which is reflected as the emission ratio of the acceptor to the donor fluorescence.

The FRET-based cameleon series (Figure 1I) were the first genetically encodable fluorescent Ca^2+^ indicators developed. The binding domain, consisted of calmodulin (CaM) and CaM-binding peptide M13, was inserted between the flanking FPs, EBFP (enhanced blue FP) with EGFP (enhanced green FP) or ECFP (enhanced cyan FP) with EYFP (enhanced yellow FP) (Miyawaki et al., 1997). The cameleon with the ECFP/EYFP pair, named as YC (yellow cameleon), was further engineered. Noticeably, the YC series were the first genetically encodable indicators tested in a living organism. In 2000, Kerr and coworkers expressed recombinant YC2.1 and YC3.1 in *Caenorhabditis elegans* and recorded the FRET signal during pharynx muscle contractions (Kerr et al., 2000). Many further engineered YC indicators have been reported, including YC 3.6, YC 4.6, YC 6.1, and YC-nano series (Truong et al., 2001; Nagai et al., 2004; Horikawa et al., 2010). YC-nano variants exhibit remarkably high sensitivity (∆*R*/*R*_0_ = 1,450%) and high Ca^2+^ affinities (*K*_d_ = 15–140 nM).

Troponin serves as the Ca^2+^-binding domain in the TN series and Twitch series of FRET-based GECIs (Heim and Griesbeck, 2004; Mank et al., 2006; Mank and Griesbeck, 2008) For these GECIs, troponin C (TnC) is inserted between a pair of CFP and YFP (Citrine). CaM used in cameleons has many native binding partners that may cause interference and make their in-cell response less substantial than their *in vitro* response. TnC, on the other hand, is only native to skeletal and cardiac muscles, and its Ca^2+^ binding can be affected by far fewer cellular binding partners. The TN series exhibit a wide range of affinities (*K*_d_ from 470 nM to 29 μM) and suitable ∆*R*/*R*_0_ values (e.g., ∼160% for TN-L15 in HEK 293 cells) (Heim and Griesbeck, 2004; Mank et al., 2006; Mank and Griesbeck, 2008). TN-XXL was later engineered with the full-length TnC replaced by a minimal domain of TnC followed by directed evolution to obtain Twitch variants with varying maximum responses and affinities (Thestrup et al., 2014). With *K*_d_ values of ∼100–200 nM, Twitch 1, Twitch 2, and Twitch 3 are suitable for lower Ca^2+^ concentration environments, such as the intracellular space. With *K*_d_ values of 280–925 μM, Twitch 4 and Twitch 5 are better-suited in higher Ca^2+^ concentration environments such as the endoplasmic reticulum.

In general, the vast majority of FRET-based GECIs use a pair of cyan (e.g., ECFP and mCerulean) and yellow (e.g., EYFP, cpVenus, and mCitrine) FPs to report FRET signal changes. The binding domains can be CaM- or TnC-based. They have been demonstrated as robust and effective tools in mammalian cells including dissociated neurons (Honarnejad et al., 2013; Thestrup et al., 2014).

#### 2.3.2 Single FP-Based Ca^2+^ Indicators

**FIGURE 2:**
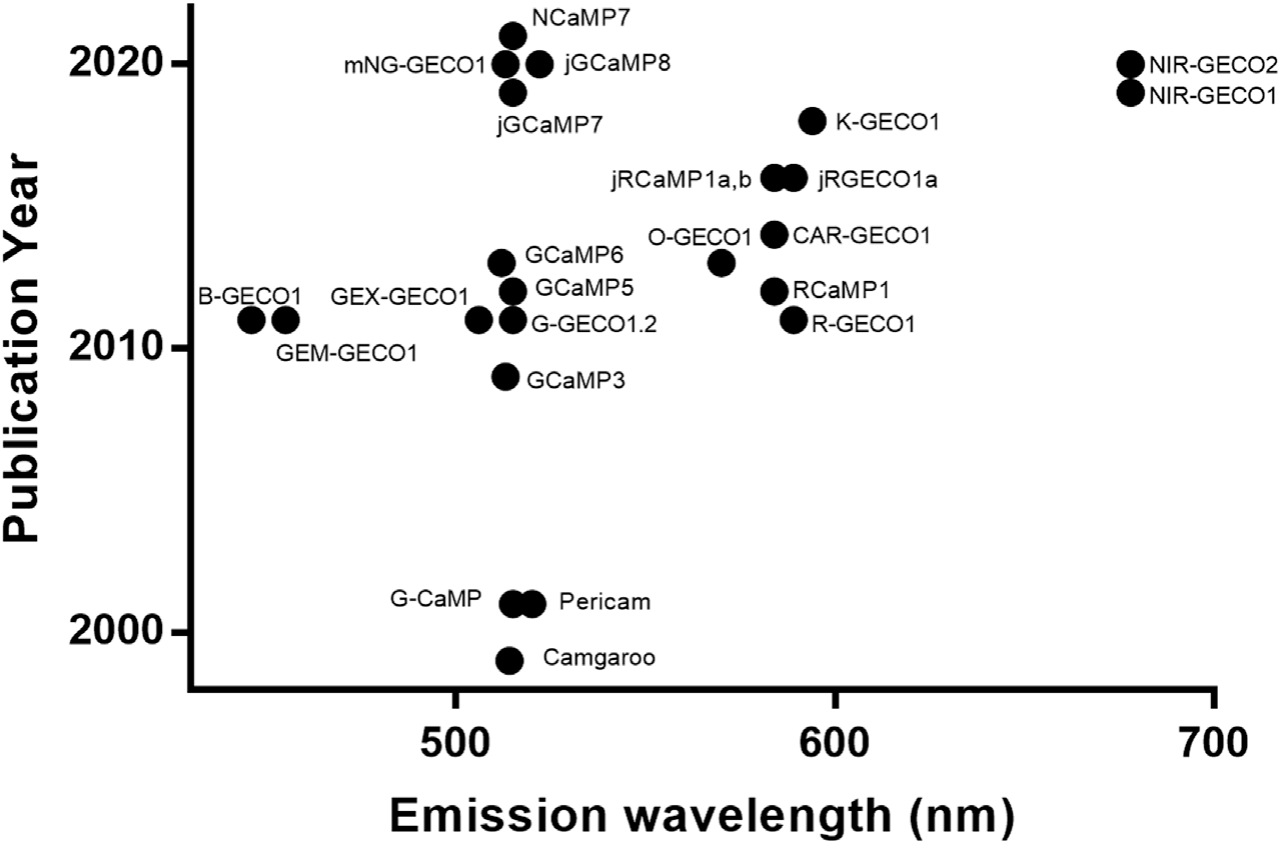
Historical overview of GECI development. Selected GECIs are plotted with *y*-axis being the publication year and *x*-axis being the peak emission wavelength. The template FPs include ones based on BFP (Zhao et al., 2011), YFP (Baird et al., 1999), GFP (Nakai et al., 2001; Tian et al., 2009; Zhao et al., 2011; Akerboom et al., 2013; Dana et al., 2019; Zhang et al., 2021), mNeonGreen (Subach et al., 2020; Zarowny et al., 2020), mApple (Zhao et al., 2011; Dana et al., 2016), mRuby (Akerboom et al., 2013; Dana et al., 2016), FusionRed (Shen et al., 2018), and mIFP (Qian et al., 2019, 2020).

Single FP-based Ca^2+^ indicators are, arguably, the crown jewels of the GECI field. Compared to FRET-based GECIs, their narrower excitation and emission spectra make them ideal for multiplexed imaging. With extensive engineering for more than 2 decades (Figure 2), they have achieved remarkably high sensitivity and colour variety. Selected single FP-based GECI are included in Supplementary Table S1.

##### 2.3.2.1 Circularly Permuted and Non-Circularly Permuted FPs For Indicator Design

**FIGURE 3:**
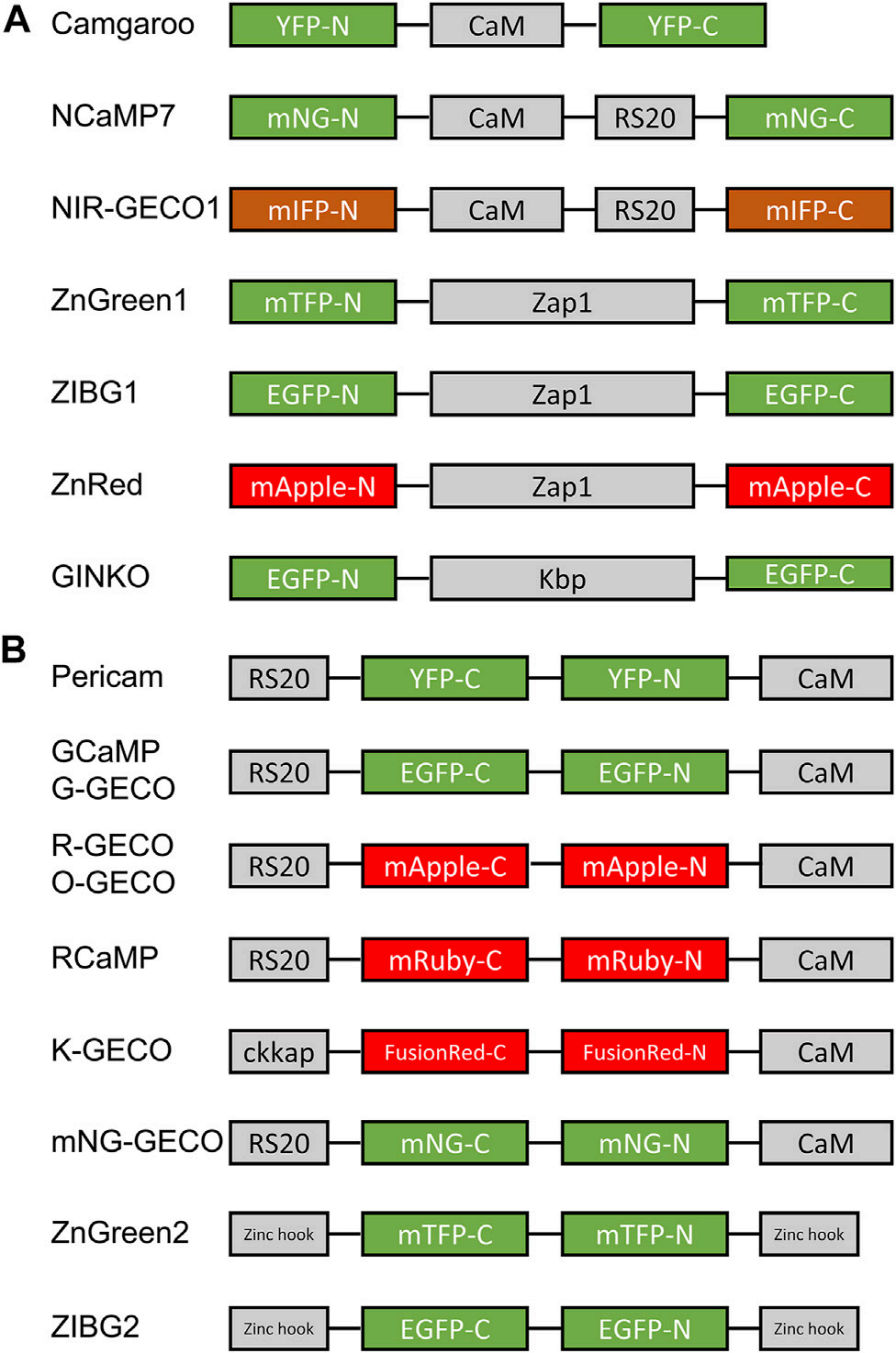
Schematic illustrations of representative cp and ncp FP-based indicators. **(A)** An ncp FP-based indicator is constructed by inserting a binding domain into an FP. Examples of ncp FP-based indicators include camgaroo (Baird et al., 1999), NCaMP7 (Subach et al., 2020), NIR-GECO1 (Qian et al., 2019), ZnGreen1 (Chen and Ai, 2016), ZIBG1 (Chen et al., 2019), ZnRed (Chen and Ai, 2016), and GINKO (Shen et al., 2019). **(B)** A cp FP-based indicator is constructed by inserting a cp FP into a binding domain or by fusing two interacting motifs to the termini of the cp FP. The cpFP indicators include pericam (Nagai et al., 2001), GCaMP (Chen et al., 2013; Dana et al., 2019), G-GECO (Zhao et al., 2011), R-GECO (Zhao et al., 2011), O-GECO (Wu et al., 2013), RCaMP (Akerboom et al., 2013), K-GECO (Zhao et al., 2011), mNG-GECO (Zarowny et al., 2020), ZnGreen2 (Chen and Ai, 2016), and ZIBG2 (Chen et al., 2019).

The two topologies for how an FP can be incorporated into an indicator are circularly permuted (cp) (Figure 3B) and non-circularly permuted (ncp) (Figure 3A) (Nasu et al., 2021). The cpFP topology involves rearrangement of the primary sequences of FPs by which the original N- and C-termini are connected *via* a new linker. New N- and C-termini are created on the β-barrel close to the phenolate group of the chromophore. An indicator based on a cpFP typically has the binding domain(s) fused to the new termini. The ncpFP-based indicators are based on the insertion of the binding domain into the primary sequence of the FP such that the binding domain is located close to the chromophore. The binding event changes the conformation of the binding domain, which subsequently influences the chromophore environment, leading to a fluorescence signal change. Here we summarize the collection of the single FP GECIs designed with cp and ncpFP topologies during the last 2 decades (Figure 3).

##### 2.3.2.2 Camgaroos

Camgaroo was the first single FP-based GECI to be developed (Baird et al., 1999). It is based on a ncp design with a CaM inserted into the YFP sequence: EYFP (1–144)-GGT-CaM-EL-EYFP (146–238). The ∆*F*/*F*_0_ for this YFP-based GECI can be as large as 6-fold and the apparent *K*_d_ of camgaroo is 7 µM.

##### 2.3.2.3 GCaMP

Green fluorescent GECIs are widely used and intensively optimized by indicator developers (Figure 1H). In 2001, Nakai and coworkers reported the first generation of GCaMP, which is a cpEGFP-based green GECI showing a positive response upon Ca^2+^ binding (∆*F*/*F*_0_ of 3.5), a Hill coefficient of 3.3, and a *K*_d_ of 235 nM (Nakai et al., 2001). The contemporaneous and similarly designed cpEYFP-based flash-pericam has a ∆*F*/*F*_0_ of 7 and a *K*_d_ of 700 nM (Nagai et al., 2001).

Initially, the first generation GECIs were not competitive to small molecule-based Ca^2+^ indicators in terms of brightness, photostability, and well-tuned affinities. This situation changed when GCaMP3 was introduced to the GECI toolbox. GCaMP3 was developed *via* structure-guided site-directed mutagenesis and semi-rational library screening. GCaMP3 is 4-fold brighter than GCaMP2, has a ∆*F*/*F*_0_ that is 3-fold that of GCaMP2, and a reduced Hill coefficient (*n*_*H*_ = 2.1) (Tallini et al., 2006; Tian et al., 2009, 3). GCaMP3 was able to provide a substantial response to a single action potential, enabling its broader utility in neuroscience. Because GECIs were prevalently applied in neuroscience, the GECI performance screening was done in dissociated neurons for the development of GCaMP6 (Chen et al., 2013), jGCaMP7 (Dana et al., 2019), and jGCaMP8 (Zhang et al., 2021). The letter “j” in the names denotes the Howard Hughes Medical Institute’s Janelia campus, where they were developed.

jGCaMP7 variants were optimized for sensitivity, kinetics, Ca^2+^ affinity, and brightness (Dana et al., 2019). jGCaMP7s (sensitive) and jGCaMP7f (fast) have improved sensitivity and kinetics for single action potential imaging. jGCaMP7s provides the biggest response to a single action potential with a 65.6% increase in fluorescence. jGCaMP7f responds rapidly to 10 action potentials with a half-rise time of 75 ms and half-decay time of 520 ms, which is comparable to GCaMP6f (a half-rise time of 80 ms and half-decay time of 335 ms) (Chen et al., 2013; Dana et al., 2019). With a 50% increase in resting fluorescence compared to GCaMP6s, jGCaMP7b (bright) is suitable for use at lower protein expression levels. jGCaMP7c (contrast) has the largest fluorescent response (∆*F*/*F*_0_ = 145) and the lowest brightness in the absence of Ca^2+^.

The major improvement for the latest jGCaMP8 series is with respect to the kinetics (Zhang et al., 2021). Variants with various CaM-binding peptides were screened for high sensitivity and fast kinetics and resulted in jGCaMP8 variants that feature a CaM-binding peptide from endothelial nitric oxide synthase. Compared to jGCaMP7, all jGCaMP8 variants have shorter rise and decay times, making them more suitable for tracing fast-paced neuronal activities. The half-rise time in response to a single action potential is 7.0 ± 0.7 ms for jGCaMP8f, which is 3-fold shorter than that for jGCaMP7f.

##### 2.3.2.4 mNeon Green-Based GECI

The brightness of GCaMPs is limited by the inherent brightness of EGFP. mNeonGreen is one of the brightest monomeric GFPs, which is 1.8-fold brighter than EGFP (Shaner et al., 2013). Zarowny *et al.* and Subach *et al.* independently developed ncp mNeonGreen-based GECIs named mNG-GECO1 and NCaMP7, respectively, (Subach et al., 2020; Zarowny et al., 2020). In the Ca^2+^-bound state, brightness of mNG-GECO1 is 59% higher than that of GCaMP6s. Its ∆*F*/*F*_0_ of 45 is comparable to that of GCaMP6 (Chen et al., 2013), but the sensitivity of mNG-GECO1 is about half of that of GCaMP6s in neurons. The brightness of NCaMP7 is 70% higher than that of GCaMP6s and ∆*F*/*F*_0_ of NCaMP7 is 89. The sensitivity of NCaMP7 is comparable to that of GCaMP6s in neurons, but with slower kinetics. Although neither mNG-GECO nor NCaMP7 surpasses GCaMP6s as a tool for neuronal activity imaging, mNeon Green-based GECIs have an enormous potential to become state-of-art indicators for neuronal imaging with further optimization.

##### 2.3.2.5 Red, Far-Red, and Near-Infrared GECI

Limited depth of imaging is one of the limitations associated with the green and blue GECIs, as biological samples absorb and scatter incoming light most strongly in this region of the spectrum. Absorption and scattering are less severe with photons of longer wavelengths (Ash et al., 2017). Thus, the red-shifted GECIs are more suitable for deeper sample imaging. Another advantage of red-shifted GECIs is their spectral compatibility with blue light-activated optogenetics tools.

R-GECO was the first reported red-fluorescent GECI and was developed using cpRFP mApple (Zhao et al., 2011). R-GECO1 exhibits a 16-fold Ca^2+^ response. The brightness of R-GECO1 is 10.2 mM^−1^cm^−1^ at its brighter Ca^2+^-bound state. Further engineering of R-GECO1 led to different variants: R-GECO1.2, O-GECO1, REX-GECO1, and CAR-GECO1 (Wu et al., 2013, 2014). R-GECO1.2 is twice as sensitive as R-GECO1 but has a larger *K*_d_ at 1,200 nM. O-GECO1 is a blue-shifted variant of R-GECO with excitation wavelengths at 543 nm (Ca^2+^ bound) and 545 nm (Ca^2+^ unbound), emission wavelengths at 564 nm (Ca^2+^ bound) and 570 nm (Ca^2+^ unbound), and an *in vitro* ∆*F*/*F*_0_ of 146. However, its weak affinity for Ca^2+^ (large *K*_d_ = 1,500 nM) made it not suitable for Ca^2+^ detection in low Ca^2+^ environments. Directed evolution on R-GECO1 led to a red-shifted variant CAR-GECO1 with peak excitation and emission wavelengths at 560 and 609 nm, respectively. CAR-GECO1 has a ∆*F*/*F*_0_ of 27 and a *K*_d_ of 490 nM *in vitro*. REX-GECO1 is a long Stokes shift red fluorescent indicator with a substantially blue-shifted excitation: the one-photon excitation is at 480 nm and the two-photon excitation is at 960 nm (Wu et al., 2014). REX-GECO1 has a ∆*R*/*R*_0_ (582–480 nm) of 100 *in vitro* and a *K*_d_ of 240 nM. Although effective, these mApple-based GECIs are all limited by photoactivation by blue light (Wu et al., 2013), preventing their utility with blue light-activated optogenetic tools.

In parallel to the development of R-GECO1, Akerboom and coworkers developed mRuby-based RCaMP series (Akerboom et al., 2013). RCaMP1f and RCaMP1h are sensitive to Ca^2+^ *in vitro* with ∆*F*/*F*_0_ values of 12.3 and 10.5, respectively. Their ∆*F*/*F*_0_ values in HEK cells are at 1.8 and 2.0, both smaller than that of R-GECO1 (∆*F*/*F*_0_ = 2.5). RCaMP1f and 1 h also exhibit larger *K*_d_ values at 1,900 and 1,300 nM, respectively. On the other hand, RCaMP1f and 1 h are brighter than R-GECO1 with a one-photon brightness of 28 and 33 mM^−1^cm^−1^, respectively, and two-photon brightness of 9.2 and 8.1 mM^−1^cm^−1^, respectively, (one-photon brightness of R-GECO1 is 10.2 mM^−1^cm^−1^; two-photon brightness of R-GECO1 is 3.8 mM^−1^cm^−1^). The lack of photoactivation of RCaMP1 enables it to be used simultaneously with the blue light-activated channelrhodopsin-2 (ChR2).

Dana and coworkers optimized the RCaMP1h and R-GECOs for neuronal imaging (Dana et al., 2016). One of the resulting GECIs, jRGECO1a, has a ∆*F*/*F*_0_ of 11.6 and a *K*_d_ at 148 nM *in vitro*. Its performance in neurons is comparable to that of GCaMP6f. However, jRGECO1a inherited the photoactivatable property of R-GECO1, preventing it from being used with blue light-activated optogenetic tools. This effort also produced jRCaMP1a and 1b, which have *K*_d_ values at 214 and 712 nM (Akerboom et al., 2013). Although neither is more sensitive than jRGECO1a, they are both 2-fold brighter than jRGECO1a and allow simultaneous photo-stimulation of ChR2 and imaging of jRCaMP1a/b.

In other work, Shen and coworkers developed a FusionRed-based GECI called K-GECO1, with less blue-light photoactivation than that of R-GECOs and a higher Ca^2+^ affinity than those of RCaMPs (Shen et al., 2018). K-GECO1 was engineered with cpFusionRed as the fluorescent reporter domain and ckkap in place of RS20 in the Ca^2+^-binding domain as previously described in RCaMP2 (Inoue et al., 2015). K-GECO1 has a *K*_d_ for Ca^2+^ at 165 nM and a brightness of 27 mM^−1^cm^−1^ (Shen et al., 2018). In neurons, K-GECO1 responds with action potentials with a better ∆*F*/*F* than most red GECIs except jRGECO1a. K-GECO1 has negligible blue-light photoactivation, enabling simultaneous imaging of K-GECO1 and photo-stimulation of optogenetic tools such as ChR2.

Despite the variety of β-barrel FPs, there are none that fluoresce well into the near-infrared region of the colour spectrum. Another class of FPs, biliverdin (BV)-binding FPs (BV-FPs), enable the development of more red-shifted GECIs by providing far-red to near-infrared fluorescence. NIR-GECO1, developed from the BV-FP mIFP, was the first reported NIR GECI. NIR-GECO1 is based on a ncp mIFP into which CaM and RS20 are inserted (Qian et al., 2019). NIR-GECO1 has an 8-fold inverse change and a *K*_d_ of 885 nM *in vitro*. The brightness of NIR-GECO1 is 3.9 mM^−1^cm^−1^ in the Ca^2+^-unbound state and 0.4 mM^−1^cm^−1^ in the Ca^2+^-bound state. The low brightness is due to the low QY inherited from its parent protein, mIFP (QY = 0.08) (Yu et al., 2015). The brightness of this BV-FP-based GECI is further limited by the amount of available BV in the tissue. When tested in the sensorimotor cortex of mice under paw stimuli, NIR-GECO1 revealed a response of only 0.3%, whereas the same stimuli could lead to a 10-fold greater fluorescence change with GCaMP6s.

Further development of NIR-GECO1 led to NIR-GECO2 and NIR-GECO2G (Qian et al., 2020). The most pronounced improvement is the higher affinities for Ca^2+^ with *K*_d_ values of 331 nM (NIR-GECO2) and 480 nM (NIR-GECO2G), in comparison to a *K*_d_ of 885 nM for NIR-GECO1. While sensitivity and brightness of the new variants remain similar to those of NIR-GECO1, the higher affinities alone are sufficient to substantially increase -∆*F*/*F* to a single action potential (16, 17, and 4.5% for NIR-GECO2, NIR-GECO2G, and NIR-GECO1). The Ca^2+^ response levels of NIR-GECO2 and NIR-GECO2G are comparable to those of GCaMP6f (19%) and jRCaMP1a (15%). However, NIR-GECO2 and 2G have relatively slow kinetics (*k*_off_ values are 3.0 s^−1^ for NIR-GECO2 and 3.7 s^−1^ for NIR-GECO2G) and low brightness (4.0 mM^−1^cm^−1^ for NIR-GECO2 and 4.5 mM^−1^cm^−1^ for NIR-GECO2G). Development of a further improved variant may require further engineering of NIR-GECO or using a brighter BV-FP.

## 3 Zn^2+^ Ion Indicators

### 3.1 The Roles of Zn^2+^ in Cell Physiology

Zinc (Zn^2+^) is an essential trace metal element in biology. Zn^2+^ is incorporated in over 300 enzymes that have a broad range of biological functions including: regulation of smell, taste, and appetite; synthesis of DNA and RNA; hormonal regulation; immune functions; and antioxidation (Frassinetti et al., 2006). Zn^2+^ is transported mainly by albumin in the blood and is maintained at a concentration of 1–10 µM in human serum or plasma (Frassinetti et al., 2006). Total cytosolic Zn^2+^ concentration is a few hundreds of micromolar, but most Zn^2+^ is tightly bound, leaving free Zn^2+^ at a picomolar level. Free Zn^2+^ is heterogeneously distributed in cell: 1 pM in the endoplasmic reticulum and the Golgi apparatus, 0.1 pM in the mitochondrial matrix, 180 pM in the cytosol, and 200 pM in the nucleus (Carter et al., 2014). Some important cellular activities are accompanied by Zn^2+^ dynamics. For example, Zn^2+^ secretion is associated with insulin secretion of human pancreatic β-cells (Chen et al., 2019). Zn^2+^ is also suggested to play important roles in the brain for its Ca^2+^-dependent release from synaptic vesicles (Assaf and Chung, 1984).

### 3.2 Small Molecule-Based Zn^2+^ Indicators

**FIGURE 4:**
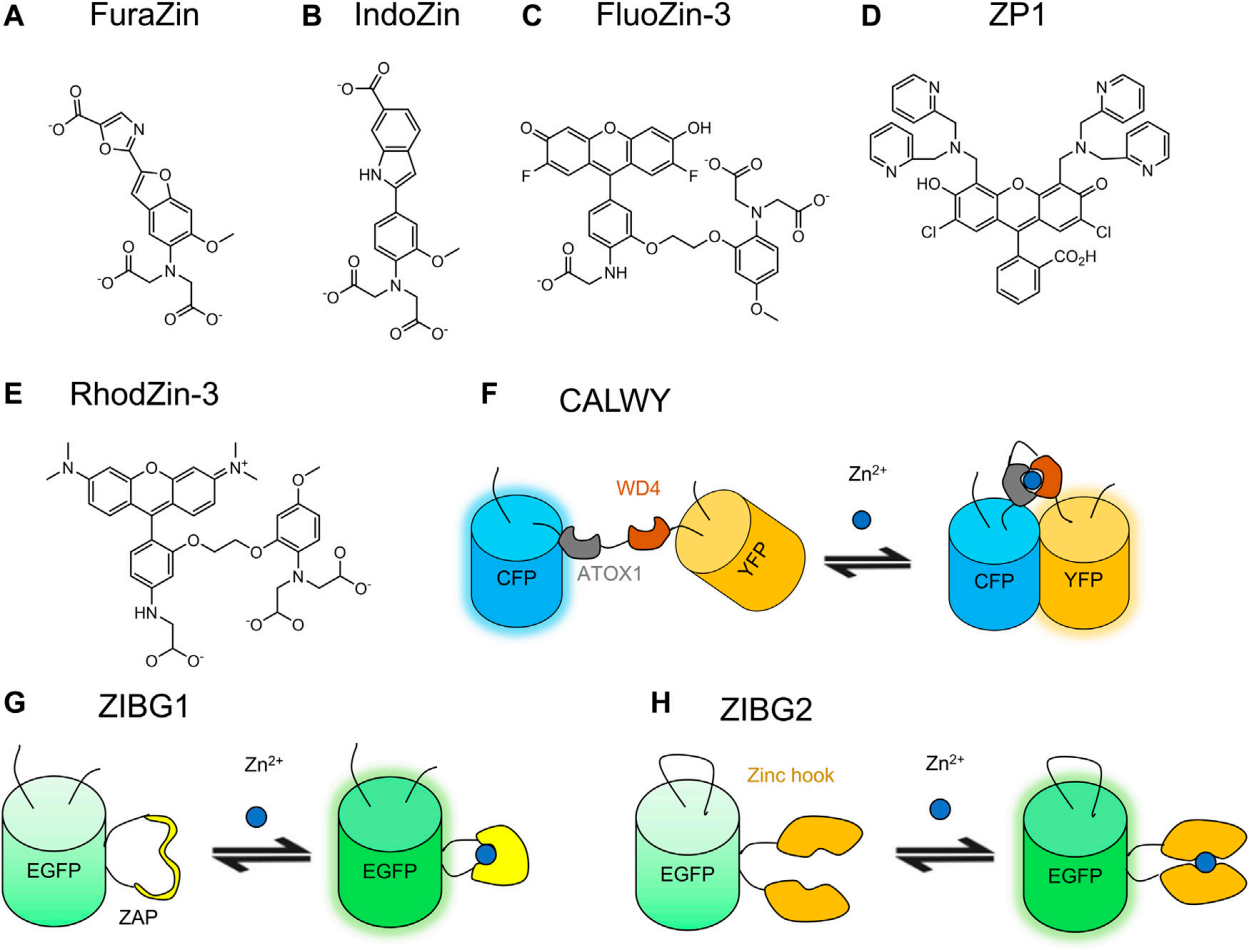
Molecular structures of selected small molecule-based Zn^2+^ indicators and schematic representations of selected GEZIs. Many small molecule-based Zn^2+^ indicators are structurally similar to small molecule-based Ca^2+^ indicators. **(A)** FuraZin, **(B)** IndoZin, and **(C)** FluoZin-3 have fluorophore moieties that are identical to those of fura-2 and indo-1, and fluo-4, respectively, (Gee et al., 2002). **(D)** ZP1 is composed of a di-2-picolylamine (DPA) Zn^2+^ chelator and a green-fluorescent fluorescein moiety (Walkup et al., 2000). **(E)** RhodZin-3 is a rhodamine-based red fluorescent Zn^2+^ indicator (Gee et al., 2002). **(F)** CALWY (Vinkenborg et al., 2009) is a FRET-based GEZI with the Zn^2+^ binding moiety of ATOX1/WD4. **(G)** ZIBG1 (Chen et al., 2019) is based on the Zn^2+^ binding ZAP1 protein and ncpEGFP. **(H)** ZIBG2 (Chen et al., 2019) is based on Zinc hook and cpEGFP.

Analogous to small molecule-based Ca^2+^ indicators, small molecule-based Zn^2+^ indicators also consist of a Zn^2+^ chelator and a fluorophore (Figures 4A–E), function using the principle of PeT, and are suitable for live-cell imaging. Common fluorophores include quinoline, fluorescein, 4-aminoapthalimide, and BODIPY (Carter et al., 2014). The small molecule-based Zn^2+^ indicators have been comprehensively reviewed previously by Carter and coworkers (Carter et al., 2014).

#### 3.2.1 BAPTA-Based Zn^2+^ Indicators

As Zn^2+^ and Ca^2+^ are both divalent cations, existing Ca^2+^ indicators provided templates for the development of Zn^2+^ indicators. By removing one or more chelating moieties on the BAPTA-based Ca^2+^ indicators, Gee and coworkers created indicators that have a weaker affinity for Ca^2+^ and a much higher affinity for Zn^2+^ (Gee et al., 2002). These indicators include FluoZins, IndoZin, FuraZin, RhodZin, X-RhodZin, and NewPort Green PDX. Among these indicators, FuraZin (Figure 4A) and IndoZin (Figure 4B) are ratiometric indicators structurally similar to their templates fura-2 and indo-1, respectively. FuraZin exhibits ratiometric excitation at 378 and 330 nm; IndoZin exhibits ratiometric emission at 480 and 390 nm. FluoZin-1 and FluoZin-3 (Figure 4C) are extremely sensitive to Zn^2+^ with a ∆*F*/*F*_0_ of 200. RhodZin (Figure 4E) and X-RhodZin also have large ∆*F*/*F*_0_ values of 150 and 100, respectively. FluoZin-3 and NewPort Green PDX are specific to Zn^2+^ with no response to Ca^2+^. The affinities of these BAPTA-based Zn^2+^ indicators for Zn^2+^ are typically in the nanomolar (e.g., *K*_d_ = 15 nM for FluoZin-3) to micromolar (e.g., *K*_d_ = 40 µM for NewPort Green PDX) range, which are not ideal for physiological Zn^2+^ concentrations that are typically at the picomolar level.

#### 3.2.2 Fluorescein-Based Zn^2+^ Indicator

Fluorescein-based Zn^2+^ indicators with other Zn^2+^-binding moiety include Zinpyr (ZP), ZnAF, Zinspy (ZS), and QZ families. ZP indicators contain a di-2-picolylamine (DPA) Zn^2+^ chelator. ZP1 (Figure 4D) is cell-permeable and has no substantial response to Ca^2+^ or Mg^2+^ (Walkup et al., 2000). It binds Zn^2+^with a ∆*F*/*F*_0_ of 3.1 and a *K*_d_ of 700 pM. The further improvement led to ZP3 with a brightness of 78 mM^−1^cm^−1^ in the Zn^2+^-bound state, a ∆*F*/*F*_0_ of 6, and a *K*_d_ of 700 pM (Chang et al., 2004a). Instead of a symmetrical fluorescein platform with two DPA groups in ZP1—3, ZP4 employs an asymmetrical fluorescein platform with a Zn^2+^ chelator consisted of one DPA and an additional aniline group (Burdette et al., 2003). ZP4 is cell permeable and exhibits a ∆*F*/*F*_0_ of 5 and a *K*_d_ of 650 pM. More ZP variants were created with modification on the electron-withdrawing groups on either the fluorophore or the chelator. ZP8, for example, has a ∆*F*/*F*_0_ of 11 and a *K*_d_ of 600 pM (Chang et al., 2004b). ZP9 and ZP10 contain a pyrrole group and an *N*-methypyrrole group, respectively, in the asymmetrical Zn^2+^ chelator, weakening their affinities substantially (the *K*_d_ of ZP9 is 690 nM and that of ZP10 is 1.9 µM) (Zhang et al., 2008).

ZnAF probes were developed by attaching a DPA moiety to different positions on the benzoic acid moiety of fluorescein. ZnAF probes have lower background fluorescence than ZP probes. Among this family, ZnAF-2, ZnAF-1F, and ZnAF-2F are highly sensitive to Zn^2+^ with ∆*F*/*F*_0_ values of 51, 69, and 60, respectively, (Hirano et al., 2000, 2002).

By using one or two pyridyl amine thioether moieties as the Zn^2+^ chelator, ZS indicators help to address the lack of specificity of ZP indicators. While ZP indicators can also bind to Fe^2+^, ZS2 and ZS4 show better specificity for Zn^2+^ over other divalent ions such as Fe^2+^, Ca^2+^, Mn^2+^, and Mg^2+^ (Nolan and Lippard, 2004). ZS1, ZS2, and ZS3 exhibit ∆*F*/*F*_0_ values in the range of 1.5–4.5. Further improvement on ZS indicators lowered the background fluorescence and improved ∆*F*/*F*_0_ by replacing the thioether with a thiophene (Nolan et al., 2006). ZSF7 has a ∆*F*/*F*_0_ of 42 and a *K*_d_ of 33 μM.

The QZ family employs an 8-aminoquinoline to bind Zn^2+^. These indicators show remarkably large ∆*F*/*F*_0_ values (∆*F*/*F*_0_ = 150 for QZ1 and ∆*F*/*F*_0_ = 120 for QZ2). With an affinity in the micromolar range, they were only suitable for the detection of Zn^2+^ dynamics at relatively high concentrations of Zn^2+^.

#### 3.2.3 Other Fluorophore-Based Zn^2+^ Indicators

Fluorophores such as boron dipyrromethene (BODIPY) and rhodamine can provide some advantages when used for the construction of Zn^2+^ indicators. BDA (Wu et al., 2005), a BODIPY-based indicator, has a low p*K*_a_ of 2.1 and is less pH sensitive than fluorescein-based indicators. BDA is a sensitive green Zn^2+^ indicator with a ∆*F*/*F*_0_ of 10.5 and a *K*_d_ of 1 nM. These properties make BDA suitable for applications in which pH is not constant. Rhodamine is a photostable red fluorescent fluorophore that has been incorporated into Zn^2+^ indicators including ZRL1 (Du and Lippard, 2010), SiR-Zn (Koide et al., 2011), and ZIGIR (Ghazvini Zadeh et al., 2020). The peak excitation and emission wavelengths of ZRL1 are at 569 and 595 nm, respectively, (Du and Lippard, 2010). SiR-Zn incorporates a silicon atom in the chromophore and is more red-shifted than other rhodamine-based indicators with the peak excitation and emission wavelengths at 650 and 665 nm, respectively, (Koide et al., 2011). The indicator is useful for intracellular Zn^2+^ detection with a nanomolar affinity and a ∆*F*/*F*_0_ of 15. ZIGIR is a red Zn^2+^ indicator that is sensitive (∆*F*/*F*_0_ > 100), cell-permeable, and granule-specific (Ghazvini Zadeh et al., 2020).

### 3.3 Genetically Encodable Zn^2+^ Indicators

The abundance of Zn^2+^-binding proteins in nature provides a number of suitable candidates for engineering GEZIs. They include the CXXC-motif-containing metal-binding domains Atox1/WD4 used in the CALWY family, yeast transcription factor Zap1 in the Zap family, and yeast transcription factor Zif268 in the Zif family.

Most GEZIs are FRET-based. ECFP and mCitrine-based ZifCY2 exhibits a ∆*R*/*R*_0_ of 4.0 but is limited by its millimolar affinity (Dittmer et al., 2009). Benefiting from the higher Zn^2+^ affinities of the Atox1/WD4 Zn^2+^-binding pair (Figure 2B), Cerulean and Citrine-based eCALWYs (Figure 4F) exhibit affinities ranging from picomolar to nanomolar (Vinkenborg et al., 2009). Zap indicators also exhibit high Zn^2+^ affinities: ECFP and mCitrine-based ZapCY1 exhibits a ∆*R*/*R*_0_ of 2.15 and a *K*_d_ of 2.5 pM; ZapCY2 exhibits a smaller ∆*R*/*R*_0_ of 1.5 and a larger *K*_d_ of 810 pM (Qin Y. et al., 2011).

The ZinCh and eZinCh indicators employ Zn^2+^-binding pockets engineered into FPs. Cerulean and Citrine-based ZinCh-1 was engineered with double mutations of Y39H and S208C on both FPs, leading to two binding sites that each consist of a pair of H39 and C208 (Evers et al., 2007). Further optimization on the binding sites led to eZinCh-2 with a ∆*R*/*R*_0_ of 4 and a *K*_d_ of 1 nM (Hessels et al., 2015). Although it also binds Pb^2+^ and Cd^2+^, eZinCh-2 is an effective intracellular GEZI for monitoring Zn^2+^ in the cytosol, the ER, the mitochondria, and vesicles.

Chen and coworkers developed single FP-based GEZIs with either Zap1 or a zinc hook as the binding domain (Chen and Ai, 2016; Chen et al., 2019). Zap1 contains two Zn^2+^ finger domains and undergoes a dramatic conformational change upon Zn^2+^ binding. The *Pyrococcus furiosus* Rad50 zinc hook peptide undergoes homodimerization upon Zn^2+^ binding. ZnGreen1, based on ncpmTFP1 and Zap1, shows superior performance relative to previous FRET-based GEZIs with a ∆*F*/*F*_0_ as large as 26.3 and a *K*_d_ of 633 pM (Chen and Ai, 2016). ZnGreen2, based on cpmTFP1 and zinc hooks, displays a ∆*F*/*F*_0_ of 8.7 and a *K*_d_ of 20 µM (Chen and Ai, 2016). One limitation of ZnGreen1 and ZnGreen2 is that they are inverse response indicators with poor photostability. To address this issue, Chen and coworkers replaced mTFP1 with EGFP to create ZIBG1 (Figure 4G) and ZIBG2 (Figure 4H) (Chen et al., 2019). ZIBG1 and ZIBG2 both respond positively to Zn^2+^ binding. ZIBG1 has a ∆*F*/*F*_0_ of 2.5 and a *K*_d_ of 2.81 µM; ZIBG2 has a ∆*F*/*F*_0_ of 7 and a *K*_d_ of 282 pM. A red fluorescent GEZI based on mApple and Zap1 was also created with a ∆*F*/*F*_0_ of 3.8 and two *K*_d_ values of 166 pM and 20 µM according to the biphasic titration curve.

GZnPs are a series of GEZIs also based on Zap1 and cpGFP (Qin et al., 2016; Fudge et al., 2018; Minckley et al., 2019). GZnP1 has a ∆*F*/*F*_0_ of 1.2 and a *K*_d_ of 34 pM. Engineering efforts on GZnP1 led to GZnP3 with a larger ∆*F*/*F*_0_ of 10 and a weaker affinity (*K*_d_ = 1.3 nM). The weakened affinity is more appropriate for Zn^2+^ released from granules.

## 4 K^+^ Indicators

### 4.1 The Roles of K^+^ in Cell Physiology

Potassium (K^+^) is one of the most abundant ions in biology. Besides its role as an electrolyte for body fluid balance, K^+^ also plays a pivotal role in excitable cells such as those in the heart and the nervous system (Humphries and Dart, 2015). In the resting state, K^+^ is sequestered into cells by the Na^+^/K^+^ pump and other transporters to maintain a large concentration gradient with the intracellular K^+^ concentration at around 150 mM and the extracellular K^+^ concentration at around 5 mM. When cells are excited, K^+^ effluxes through a variety of voltage-gated K^+^ channels to repolarize cells after depolarization. The dynamics of K^+^ fluxes reflect neuronal and cardiac signal propagation and therefore is an intriguing target for live-cell imaging applications.

### 4.2 Small Molecule-Based K^+^ Indicator

**FIGURE 5:**
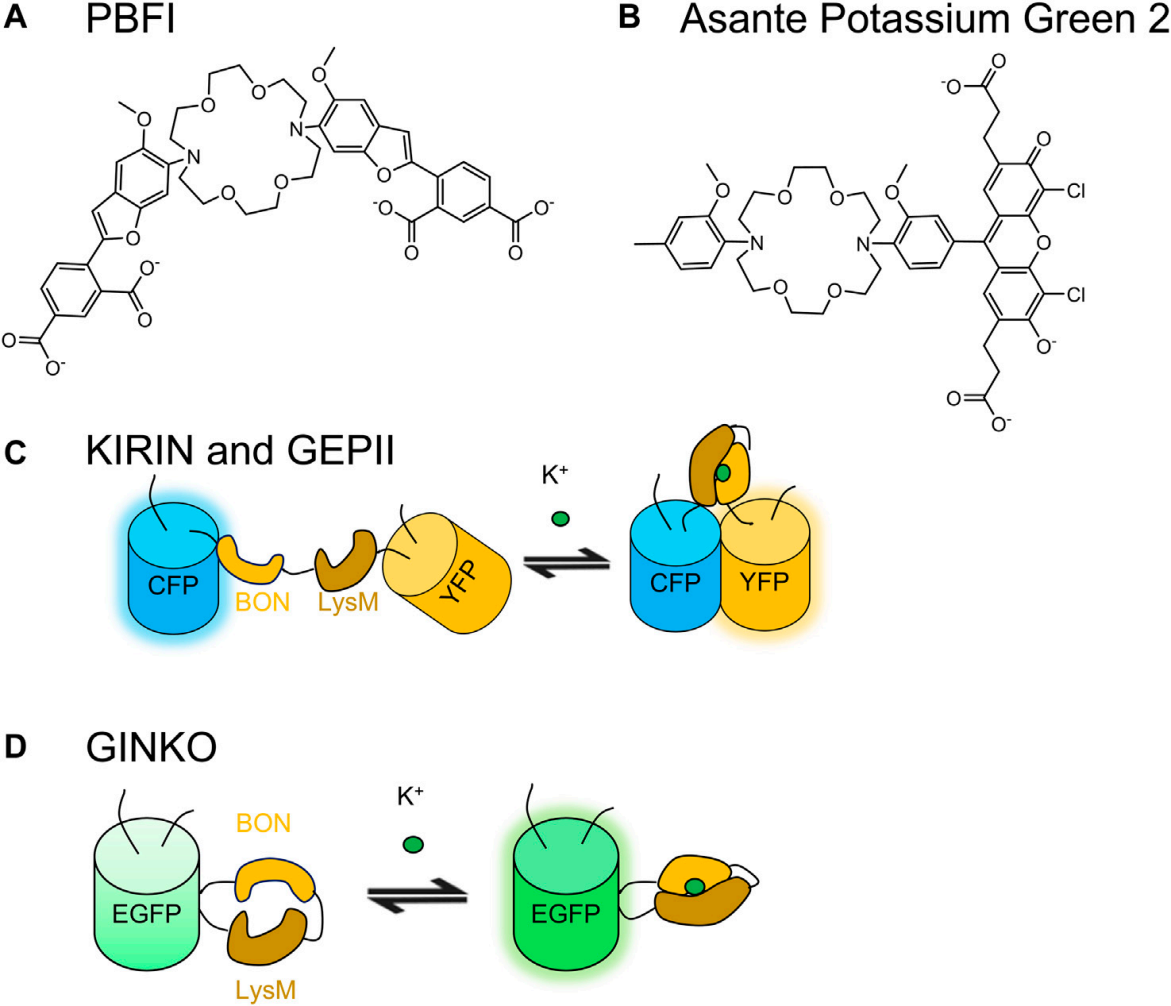
Molecular structures of selected small molecule-based K^+^ indicators (PBFI and Potassium Green 2) and schematic representations of selected GEKIs. Small molecule-based K^+^ indicators **(A)** PBFI and **(B)** Asante Potassium Green 2 (APG-2) are based on 1, 10-diaza-4, 7, 13, 16-tetraoxacyclopentadecane (Minta and Tsien, 1989; Rimmele and Chatton, 2014). **(C)** FRET-based KIRIN and GEPII are based on Kbp that consists of a BON and a LysM domain (Ashraf et al., 2016; Shen et al., 2019). **(D)** GINKO1 and GINKO2 are based on Kbp and ncpEGFP (Shen et al., 2019; Wu et al., 2021).

Potassium binding benzofuran isophthalate (PBFI) is the earliest small molecule-based K^+^ indicators (Figure 5A) (Minta and Tsien, 1989). It is excited by UV light at 350 nm in the K^+^-unbound state and 344 nm in the K^+^-bound state. Although the indicator is more specific for K^+^ (*K*_d_ = 8 mM) than Na^+^ (*K*_d_ = 21 mM), better specificity is required for cellular imaging to avoid signal interference by Na^+^, which is typically at an intracellular concentration of 10–40 mM (Minta and Tsien, 1989). PBFI exhibits low brightness primarily due to its low QY of 0.024 in the K^+^-unbound state and 0.072 in the K^+^-bound state. Asante Potassium Green-1 (APG-1) is more red-shifted than PBFI with the peak excitation wavelength at 515 nm and the peak emission wavelength at 540 nm (Rimmele and Chatton, 2014). APG-1 responds to K^+^ with a maximum 4-fold change. Improved APG-2 (Figure 5B) and APG-4 were applied in permeabilized Jurkat and U937 cells to visualize changes in intracellular K^+^ concentration (Rana et al., 2019). Recently, Ratiometric Potassium Sensor-1 (RPS-1) was developed with dual fluorophores: a K^+^-responsive fluorophore Potassium Sensor 525 (PS525) and a non-responsive Coumarin 343 for internal calibration in living tissues (Wang et al., 2021). The dye displays a ∆*F*/*F*_0_ of 6 and an apparent *K*_d_ of 137 mM.

### 4.3 Genetically Encodable K^+^ Indicators

The identification of K^+^ binding protein (Kbp) enabled the development of GEKIs (Ashraf et al., 2016). Bischof *et al.* and Shen *et al.* developed FRET-based GEKIs named GEPII and KIRIN, respectively, (Figure 5C) (Ashraf et al., 2016; Shen et al., 2019; Bischof et al., 2019). The two indicators show comparable ∆*R*/*R*_0_ and affinity values: the mCerulean3 and cpVenus-based KIRIN has a ∆*R*/*R*_0_ of 1.5 and a *K*_d_ of 1.66 mM, and the mseCFP and cpVenus-based GEPII has a ∆*R*/*R*_0_ of 2.2 and a *K*_d_ of 0.42 mM. Both FRET indicators exhibit outstanding specificity for K^+^, showing no response to Na^+^ at physiologically relevant concentrations. In addition to the FRET-based GEKIs, Shen *et al.* also described ncpGFP-based GINKO1 (Figure 5D). GINKO1 exhibits a ∆*F*/*F*_0_ of 1.5 and higher specificity for K^+^ (*K*_d_ = 0.42 mM) than Na^+^ (*K*_d_ = 153 mM). Further engineering with site-directed mutagenesis and directed evolution led to GINKO2 with better sensitivity (∆*F*/*F*_0_ = 14) and specificity (no response to Na^+^ up to 150 mM) (Wu et al., 2021).

## 5 Mg^2+^ Indicators

### 5.1 The Roles of Mg^2+^ in Cell Physiology

Mg^2+^ plays many pivotal roles in cellular processes and functions. Mg^2+^ serves as an important cofactor for almost every enzyme that needs ATP for catalysis; stabilizes nucleic acids through electrostatic interactions for DNA and RNA synthesis and repair; and regulates mitochondrial Ca^2+^ transport, voltage-gated Ca^2+^ channels, and voltage-gated K^+^ channels as a Ca^2+^ antagonist (Pilchova et al., 2017). Most intracellular Mg^2+^ is bound to biomacromolecules, such that total intracellular concentration of total Mg^2+^ is 17–20 mM but only 0.25–1.5 mM is free Mg^2+^ (Grubbs, 2002; Romani, 2013). Cells maintain a steady-state Mg^2+^ concentration through transporters such as Mrs2p, which transports Mg^2+^ into mitochondria for Mg^2+^ sequestration (Kolisek et al., 2003).

### 5.2 Small Molecule-Based Mg^2+^ Indicators

**FIGURE 6:**
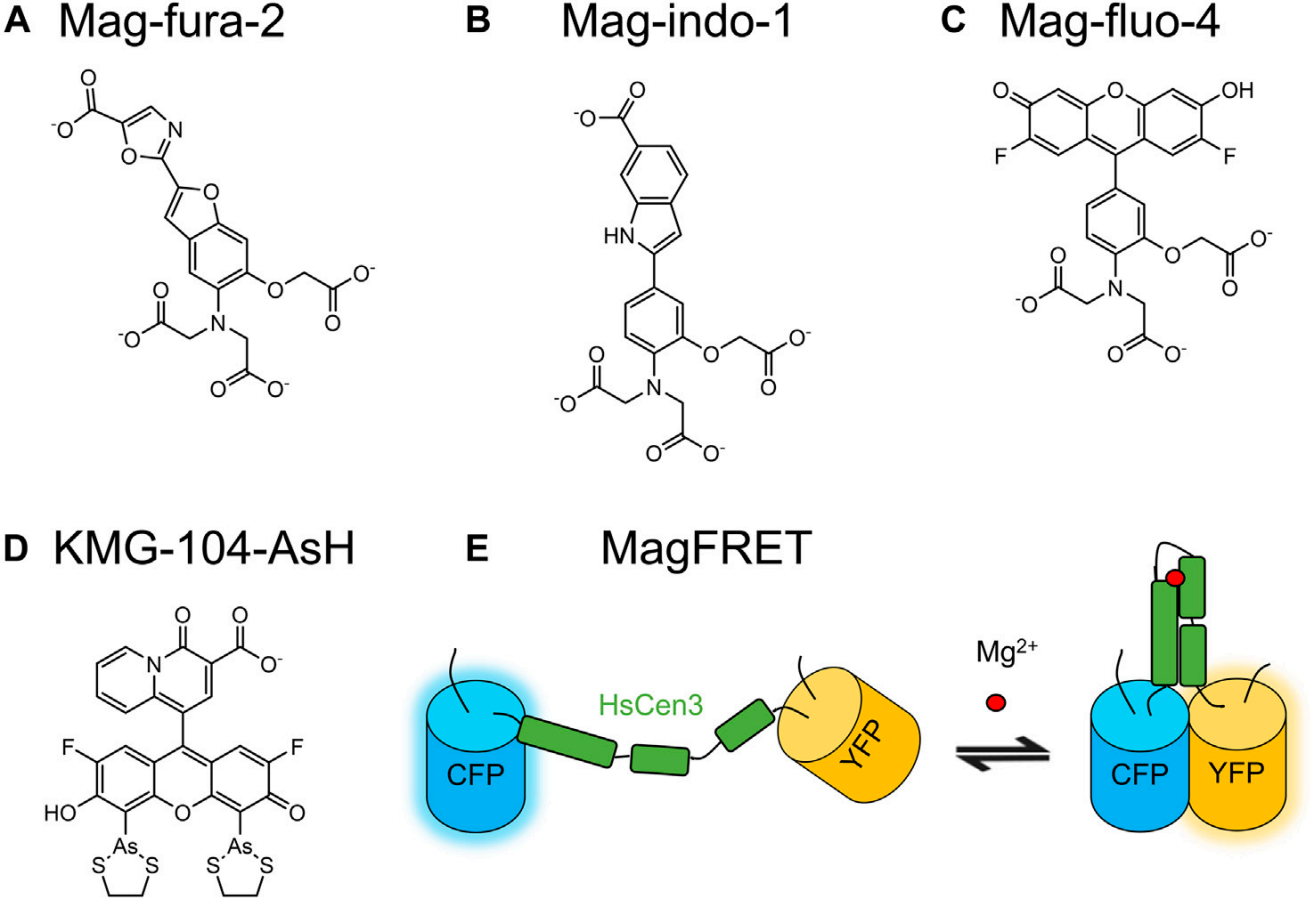
Molecular structures of selected small molecule-based Mg^2+^ indicators and schematic representation of a selected GEMI. **(A)** Mag-fura-2 (Raju et al., 1989), **(B)** Mag-indo-1 (Morelle et al., 1994), and **(C)** Mag-fluo-4 (Shmigol et al., 2001) are based on fluorophore moieties that are identical to those of the BAPTA-based Ca^2+^ indicators fura-2, indo-1, and fluo-4, respectively. As a Mg^2+^-binding moiety, they employ *o*-aminophenol-*N*, *N*, *O*-triacetic acid (APTRA) for Mg^2+^-binding. **(D)** KMG-104-AsH is a FlAsH-type indicator based on a β-diketone Mg^2+^-binding moiety (Fujii et al., 2014). KMG-104-AsH can be genetically targeted *via* covalent linking to tetracysteine-tagged proteins through its biarsenical groups. **(E)** MagFRET is a FRET-based genetically encodable Mg^2+^ indicator with the Mg^2+^ binding domain from human centrin HsCen3.

Many small molecule-based Ca^2+^ indicators that exhibit an affinity for Mg^2+^ were modified into Mg^2+^ indicators with *o*-aminophenol-*N*, *N*, *O*-triacetic acid (APTRA) as the binding group. Some examples include Mag-fura-2 (Figure 6A), Mag-indo-1 (Figure 6B), and Magnesium Green (Supplementary Table S2) (Raju et al., 1989; Morelle et al., 1994; Zhao et al., 1996). Mag-fura-2 is excited at 369 nm and Mag-indo-1 is excited at 349 nm (Raju et al., 1989). Due to the phototoxicity associated with UV excitation, Mg^2+^ indicators with excitation at longer wavelengths were developed. The green indicators Magnesium Green and Mag-fluo-4 (Figure 6C) are excited at 506 and 490 nm, respectively, (Zhao et al., 1996; Shmigol et al., 2001). The above indicators are all subject to interference from Ca^2+^ in live-cell imaging due to their millimolar affinities for Mg^2+^ and micromolar affinities for Ca^2+^.

Other binding motifs were sought to address the lack of Mg^2+^ specificity over Ca^2+^ observed with APTRA. One such motif is the charged β-diketone, which is used in KMG-103 (*K*_d_ = 1.8 mM for Mg^2+^, *K*_d_ = 6.3 mM for Ca^2+^) and KMG-104 (*K*_d_ = 2.1 for Mg^2+^, *K*_d_ = 7.5 mM for Ca^2+^) that bind Mg^2+^ preferentially (Komatsu et al., 2004). With an addition of a biarsenical structure to KMG-104, the FlAsH-based KMG-104-AsH (Figure 6D) exhibits an outstanding specificity with *K*_d_ values of 1.7 mM for Mg^2+^ and 100 mM for Ca^2+^ (Fujii et al., 2014). The FlAsH-based indicator remain quenched until it covalently links to a tetracysteine tagged protein through its biarsenical motif. This mechanism allows subcellular targeting and decreases background fluorescence. KMG-104-AsH is pH stable in the range of pH 5–6.5 and relatively photostable. The affinity, specificity and stability of KMG-104-AsH make it more suitable for live-cell Mg^2+^ imaging than APTRA-based indicators.

### 5.3 Genetically Encodable Mg^2+^ Indicators

As most Mg^2+^-binding proteins also bind Ca^2+^ with a higher affinity, one of the main challenges for developing GEMIs was to first identify a Mg^2+^-specific binding protein. The first reported GEMIs, the MagFRET series, were based on the Mg^2+^-binding protein human centrin HsCen3 and FPs Cerulean and Citrine (Figure 6E) (Lindenburg et al., 2013). HsCen3 contains two binding sites that bind both Mg^2+^ and Ca^2+^. MagFRET1 has a ∆*R*/*R*_0_ of 0.49 for Mg^2+^ and a ∆*R*/*R*_0_ of 0.19 for Ca^2+^; MagFRET2 has a ∆*R*/*R*_0_ of 0.33 for Mg^2+^ and a ∆*R*/*R*_0_ of 0.031 for Ca^2+^. MagFRET1, 2, 7, and 8 have suitable affinities for detecting Mg^2+^ (*K*_d_ = 0.15–0.89 mM) in the physiological concentration range of Mg^2+^, but they all have high affinities for Ca^2+^ (*K*_d_ = 10–57 µM).

MagIC is a ratiometric GEMI based on mCherry and Mg^2+^/Ca^2+^ sensitive cpVenus (Koldenkova et al., 2015). *K*_d_ values of MagIC are 5.1 mM for Mg^2+^ and 4.8 mM for Ca^2+^. Its affinity for Mg^2+^ is too low for detection of intracellular Mg^2+^. The indicator has an *in vitro* ∆*R*/*R*_0_ of 0.5 for Mg^2+^, which is similar to that for Ca^2+^. Just like MagFRETs, the sensitivity of MagIC to Ca^2+^ prevent it from being more widely applied. MARIO1 is another FRET-based GEMI based on a modified cytosolic Mg^2+^-sensing domain of the *E. coli* Mg^2+^ transporter CorA (CorA-CD) (Maeshima et al., 2018). MARIO1 show improved sensitivity (∆*R*/*R*_0_ = 1.53 for Mg^2+^) but still lacks specificity (*K*_d_ = 6.2 mM for Ca^2+^ and *K*_d_ = 7.2 mM for Mg^2+^).

## 6 Na^+^ Indicators

### 6.1 The Role of Na^+^ in Cell Physiology

As another one of the most abundant ions in biology, Na^+^ has many important roles and functions. It regulates osmotic pressure and fluid balance (Reynolds et al., 2006). Na^+^ also plays roles in the immune system by enhancing immune responses for pathogen clearance and affecting the differentiation and functions of immune cells (Wilck et al., 2019). In the nervous system, Na^+^ influxes during the depolarization phase of action potentials for signal propagation (Chahine and O’Leary, 2014). Na^+^ concentration is around 10 mM intracellularly and over 100 mM extracellularly (Strazzullo and Leclercq, 2014). The large gradient across the membrane is maintained by the Na^+^/K^+^ pump, which provides energy for the translocation of Ca^2+^ and other neurotransmitters. There are several small molecule-based Na^+^ indicators, but no genetically encodable indicators have been reported to date due to the lack of an appropriate Na^+^-binding protein.

### 6.2 Small Molecule-Based Na^+^ Indicators

**FIGURE 7:**
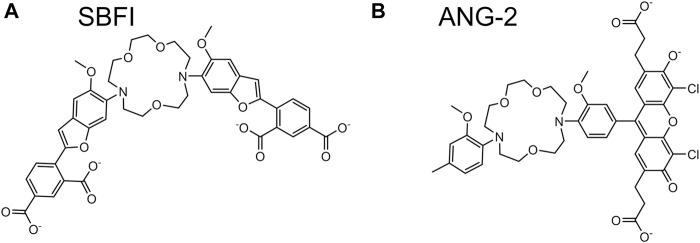
Molecular structures of selected small molecule-based Na^+^ indicators. Small molecule-based Na^+^ indicators differ from K^+^ indicators with respect to their crown ether moieties. Na^+^ indicators are typically based on 1, 7-diaza-4, 10, 13-trioxacyclopentadecane as represented for **(A)** SBFI and **(B)** ANG-2 (Minta and Tsien, 1989; Roder and Hille, 2014).

Sodium binding benzofuran isophthalate (SBFI) (Figure 7A) was developed at the same time as PBFI (Figure 6). SBFI has a *K*_d_ of 7.4 mM for Na^+^ and a *K*_d_ of 166 mM for K^+^ (Minta and Tsien, 1989). The indicator provides a ∆*F*/*F*_0_ of 2. A drawback of SBFI is its low brightness attributed to its low QY of 0.045 in the Na^+^-unbound state and 0.083 in the Na^+^-bound state. Sodium Green has visible light excitation and brighter fluorescence that enable its usage in flow cytometry (Amorino and Fox, 1995). CoroNa Green has a peak excitation wavelength at 492 nm and a peak emission wavelength at 516 nm (Meier et al., 2006; Iamshanova et al., 2016). NaTRIUM Green 2 (ANG-2) (Figure 7B) is slightly red-shifted with peak excitation and emission wavelengths at 517 and 542 nm, respectively, (Roder and Hille, 2014; Iamshanova et al., 2016). The ∆*F*/*F*_0_ value of CoroNa Green is 4 and that of ANG-2 is 20 (Iamshanova et al., 2016). The *K*_d_ value of CoroNa Green for Na^+^ is ∼80 mM and that of ANG-2 is 34 mM in presence of K^+^ and 20 mM in absence of K^+^. SBFI, CoroNa Green and ANG-2 have been used to detect changes in intracellular Na^+^ concentrations in prostate cancer cell lines (Iamshanova et al., 2016).

## 7 pH (H^+^) Indicators

### 7.1 Importance of pH in Cell Physiology

Proton (H^+^) concentration is measured as pH (−log[H^+^]). The pH values vary in different cellular compartments: 7.0–7.4 in the cytoplasm, 7.2 for ER, 6.4 in the Golgi apparatus, 5.0 in lysosomes, 5.4 in secretory granules, 6.2 in early endosomes, and 5.3 in late endosomes (Futai et al., 2000). The pH values of organelles are maintained mainly by vacuolar H^+^-ATPase (V-ATPase), which actively pumps H^+^ against its concentration gradient with the energy from ATP hydrolysis (Nelson et al., 2000). The pH environment is important for the functions of the organelles. For example, the lysosomal low pH is important for the optimal functions of lysosomal enzymes such as lysozyme. In other cases, proton gradients across organelle membrane or plasma membrane are used to provide energy for translocation of other molecules, such as neurotransmitters accumulated into synaptic vesicles (Farsi et al., 2017). In mitochondria, proton gradients provide energy for ATP synthesis (Reid et al., 1966). The capability provided by pH indicators (Supplementary Table S3), to measure pH in subcellular compartments of live cells, enables investigations of pH-related processes such as endocytosis and vesicle secretion.

### 7.2 Small Molecule-Based pH Indicators

Tsien and coworkers developed 2', 7'-bis (carboxyethyl)-5,6-carboxyfluorescein (BCECF), one of the first fluorescent small molecule-based pH indicators (Rink et al., 1982). BCECF has a p*K*_a_ at 6.98, ideal for intracellular pH measurement. The dye shows two peaks in the excitation spectrum at ∼505 and ∼470 nm. The emission intensity exhibits a 3-fold increase from pH 6.4 to pH 7.5. The isosbestic excitation wavelength of BCECF is at 440 nm, where the excitation would lead to a pH-independent emission. The seminaphtharhodafluor (SNARF) family is an alternative pH indicator series with dual excitation and dual emission. SNARF-1, for example, has two peak excitation wavelengths in an acidic environment at 515 and 544 nm, and a peak excitation wavelength of 573 nm in a basic environment (Whitaker et al., 1991). The peak emission wavelength shifts from 573 to 631 nm as pH increases. With yellow to orange fluorescence, SNARF series can be co-imaged in live cells with blue fluorescent dyes such as fura-2 (Martinez-Zaguilan et al., 1991). SNARF-1 and SNARF-2 both have p*K*_a_ values above 7 (7.6 for SNARF-1, 7.5 for SNARF-2). SNARF-4 has a lower p*K*_a_ of 6.4 to enable pH detection in slightly acidic environments (Marcotte and Brouwer, 2005). The pH indicators pHrodo green and red (Thermo-Fisher) are ideal indicators to image endocytosis because of their low fluorescence signal at neutral pH and increased fluorescence at acidic pH (Godfrey et al., 2008; Lindner et al., 2020).

### 7.3 Genetically Encodable pH Indicators

The development of many of the genetically encodable pH indicators was inspired and enabled by the intrinsic pH sensitivity of FPs themselves. The environmental pH can influence the protonated state of the chromophore, leading to fluorescence changes. EGFP shows dual excitation at approximately 390 and 480 nm and the ratio of the two peaks in the excitation spectra change in response to changes in pH (Kneen et al., 1998). When excited at 480 nm, EGFP shows an emission increases with increasing pH. EYFP (p*K*_a_ = 7.1) is more suitable for intracellular pH sensing than EGFP (p*K*_a_ = 6.15) due to its neutral p*K*_a_ (Llopis et al., 1998).

Fusing a pH-sensitive FP to a FRET donor or acceptor leads to a FRET-based pH indicator. For example, the FluBpH series is based on a fusion of EcFbFP, a flavin mononucleotide (FMN)-binding FP excited at 380 nm, and YFPs with different p*K*_a_ values, to generate FRET indicators covering different ranges of pH (Rupprecht et al., 2017). The YFPs include: Citrine (p*K*_a_ at 5.7), EYFP (p*K*_a_ at 6.1), and EYFP-H148G (p*K*_a_ at 7.5). The ratio of 570 nm (YFP fluorescence emission) to 495 nm (EcFbFP fluorescence emission) reflects the pH of the environment. Alternatively, a CFP and YFP-based pH indicator has a peak excitation wavelength at 410 nm and peak emission wavelengths at 476 nm (CFP) and 535 nm (YFP) (Hellwig et al., 2004). pHusion is a tandem concatenation of mRFP1 and the pH-sensitive EGFP (Gjetting et al., 2012). pH-Lemon, based on mTurquoise2 and EYFP, exhibits a highly sensitive ratio change in the range of pH 4.0–7.0 (Burgstaller et al., 2019).

Engineering of GFP for increased pH sensitivity has resulted in a variety of green pH indicators. One example is the ratiometric pH-sensitive variant E^2^GFP (GFP with F64L/S65T/T203Y/L231H) with a p*K*_a_ around 7 (Bizzarri et al., 2006). E^2^GFP shows ratiometric excitation and emission: the peak emission wavelength is at 510 nm at low pH and red shifts to 523 nm at high pH. In addition, the peak excitation wavelength is at 424 nm at low pH and becomes two peaks (401 and 515 nm) at high pH. Other engineering efforts resulted in the deGFPs that have a variety of p*K*_a_ values: deGFP1 variant (S65T/H148G/T203C, p*K*_a_∼8.0), deGFP2 (S65T/C48S/H148C, p*K*_a_∼7.2), deGFP3 (S65T/T203C, p*K*_a_∼6.9), and deGFP4 (S65T/C48S/H148C/T203C, p*K*_a_∼7.3) (Hanson et al., 2002).

Miesenböck and coworkers developed ratiometric pHluorin and ecliptic pHluorin by mutating key residues (Q94, R96, H148, I167, T203, S205, and E222) that affect Y66 protonation or excitation spectrum (Miesenböck et al., 1998). With a fixed emission wavelength at 508 nm, ratiometric pHluorin exhibits an excitation ratio (395–475 nm) change greater than 3-fold in the pH range from 5.5 to 7.5. Ecliptic pHluorin has a ∼5-fold fluorescence emission change in the pH range from 5.5 to 7.5. Superecliptic pHluorin introduced two mutations (F64L and S65T) on ecliptic pHluorin that led to better protein folding and improved sensitivity (∼50-fold) required for imaging of neuronal synaptic vesicle fusion and neurotransmitter release (Sankaranarayanan et al., 2000).

In addition to EGFP, other FPs have been utilized to generate a palette of pH indicators with different colours. Introducing H148G to YFP led to pHVenus with a p*K*_a_ at 7.3 (Wachter et al., 1998). pHRed is a red pH indicator based on mKeima with a p*K*_a_ of 6.5 and a ratiometric change (585 nm *versus* 440 nm) over 10-fold (Tantama et al., 2011). pHTomato is another red pH indicator with peak excitation and emission wavelengths at 550 and 580 nm, respectively, (Li and Tsien, 2012). It has a higher p*K*_a_ at 7.8 and a ∆*F*/*F*_0_ over 1 in the range of pH 7.5–9.8. pHuji is an mApple-based pH indicator with a ∆*F*/*F*_0_ of 21 in the range of pH 5.5–7.5 (Shen et al., 2014). pHmScarlet, the latest addition to red pH indicators, is 6-fold brighter than pHuji (Liu et al., 2021). As described by Shen et al., a general limitation of red fluorescent pH indicators, with respect to their use for imaging of vesicle fusion, is their lower Hill coefficients (n_H_) relative to green fluorescent pH indicators (Shen et al., 2014).

## 8 Cl^−^ Indicators

### 8.1 The Roles of Cl^−^ in Cell Physiology

Chloride (Cl^−^) is an important anion in biology. It regulates proteins and genes including kinases (e.g., Cl^−^-dependent GTP-utilizing protein kinase, nucleoside diphosphate kinase (NDPK), and with-no-lysine kinase (WNK)), channels (e.g., Na^+^-K^+^-2Cl^−^ cotransporter), and receptors (e.g., glutamate ionotropic receptor kainate (GRIK)) (Valdivieso and Santa-Coloma, 2019). Cl^−^ in mammalian cells is maintained at concentrations ranging from 5 to 100 mM by transporters such as potassium chloride cotransporter 2 (KCC2). The serum Cl^−^ concentration is maintained at 100 mM by the kidney. The gradient is used by chloride channels for cellular processes. For example, GABA type A receptor (GABA_A_) conducts Cl^−^ upon GABA binding to allow signal propagations (Goetz et al., 2007).

### 8.2 Small Molecule-Based Cl^−^ Indicators

Small molecule-based Cl^−^ indicators include 6-methoxy-*N*-(3-sulfopropyl) quinolinium (SPQ), *N*-(ethoxycarbonylmethyl)-6-methoxyquinolinium bromide(MQAE),6-methoxy-*N*-ethylquinolinium iodide (MEQ), and lucigenin. These Cl^−^ indicators are quenched by Cl^−^ through collisional quenching. The rate of fluorescence quenching is accelerated by higher concentrations of Cl^−^. All of them are long Stoke shift dyes that are excited by UV lights and emit photons at wavelengths approximately 100 nm longer than their peak excitation wavelengths (Verkman et al., 1989; Biwersi et al., 1994). When applied in cells, SPQ and MQAE show severe photobleaching (Krapf et al., 1988; Verkman et al., 1989). MEQ is much less prone to photobleaching than MQAE in Swiss 3T3 fibroblasts (Biwersi and Verkman, 1991). Lucigenin is incompatible with intracellular imaging because its electron-deficient acridine ring is prone to nucleophilic attack in biological samples (Biwersi et al., 1994).

#### 8.2.1 Genetically Encodable Cl^−^ Indicators

YFP is intrinsically sensitive to Cl^−^ (Supplementary Table S4) (Wachter and James Remington, 1999). Its fluorescence is decreased by 40% with 150 mM Cl^−^ at pH 7.0. Cl^−^ binds near the chromophore and the binding destabilizes the deprotonated form of the chromophore due to charge repulsion. This also leads to elevated p*K*_a_ (5.2 without Cl^−^, and 7.0 with Cl^−^). T203Y is a key mutation for Cl^−^ binding presented in many YFP variants (Wachter and James Remington, 1999; Arosio et al., 2007). Further engineering led to better Cl^−^ indicator variants. For example, the H148Q mutation decreases the *K*_d_ from 777 to 100 mM and increases the fluorescence response to a 50% reduction in fluorescence in presence of Cl^−^ (Jayaraman et al., 2000). I152L was introduced to reduce the *K*_d_ to 85 mM for Cl^−^ (Galietta et al., 2001). A further engineered variant mCl-YFP, with eight mutations relative to EYFP, has a higher affinity and a reduced pH sensitivity (Zhong and Schleifenbaum, 2019). These Cl^−^ sensitive FPs also respond to other halides.

Kuner and Augustine developed the first ratiometric Cl^−^ indicator, Clomeleon, based on fusion of CFP to Cl^−^-sensitive YFP (Kuner and Augustine, 2000). Clomeleon undergoes a near 80% decrease of *F*_527nm_/*F*_485nm_ ratio in presence of Cl^−^ *in vitro* and has a maximum 50% decrease in hippocampal neurons. Clomeleon has a *K*_d_ of 167 mM for Cl^−^, leading to relatively small responses in physiologically relevant conditions. To decrease the *K*_d_, H148Q, I152L, and V163S were introduced to Clomeleon and that reduced the *K*_d_ to ∼30 mM (Markova et al., 2008). SuperClomeleon was engineered by changing in the linker between two FPs, replacing Cerulean with CFP, and introducing other beneficial mutations (S30R, Q69T and V163A) (Grimley et al., 2013). This improved indicator exhibits a dynamic range (−∆*R*/*R*_0_) of 90% and a *K*_d_ of 8.1 mM.

ClopHensor is a dual indicator for pH and Cl^−^ that addresses complications arising from the pH sensitivity of YFP (Arosio et al., 2010). The indicator is a fusion of pH sensing E^2^GFP and a DsRed monomer. E^2^GFP can be excited at 488 nm for pH dependent Cl^−^ measurement or at 458 nm for pH-independent Cl^−^ measurement. The wavelength of 458 nm is an isosbestic point based on the pH titration profile of E^2^GFP. DsRed is excited at 543 nm with no overlapping signal from E^2^GFP. Its signal is affected by neither pH nor Cl^−^ concentrations. Hence, measurements with excitation at 458 nm, 488 nm, and 548 nm allow simultaneous measurement of pH and Cl^−^.

Other FPs have been explored as alternative Cl^−^ indicators. The jellyfish *Phialidium sp* phiYFP was identified as a naturally occurring Cl^−^-sensitive ratiometric indicator (Tutol et al., 2019b). phiYFP has two peak excitation wavelengths at 400 and 480 nm and a peak emission wavelength at 540 nm. When excited at 480 nm, the fluorescence of phiYFP decreases modestly as Cl^−^ concentration increases. When excited at 400 nm, phiYFP is a turn-on indicator with a ∆*F*/*F*_0_ of 2.5 in response to 400 mM Cl^−^. Recently, mNeonGreen was identified as another turn-on Cl^−^ indicator with a ∆*F*/*F*_0_ of 20-fold, a *K*_d_ of 9.8 mM, but also a strong pH dependency that may limit its applications (Tutol et al., 2019a).

## 9 Cu^+^ Indicators

### 9.1 The Roles of Cu^+^ in Cell Physiology

Copper is an essential nutrient to human health. It has implications in cardiovascular (Saari, 2000), immune (Percival, 1998), and nervous systems (Opazo et al., 2014). The element exists predominantly in two forms in biology: the monovalent Cu^+^ and the divalent Cu^2+^. The two different oxidation states of copper are exploited by enzymes that catalyze redox reactions (e.g., cytochrome c oxidase and NADH dehydrogenase) (Festa and Thiele, 2011). Many of these enzymes are located on the membranes of compartmented organelles in eukaryotic cells. Mitochondria, in particular, contain many copper-dependent enzymes for energy production (Baker et al., 2017). Most copper ions are in the cofactors of enzymes, leaving only 10^−18^—10^−13^ M free Cu^+^ (Tapiero et al., 2003). Excess copper is toxic because free Cu^+^ can generate free radicals in cells and destabilize iron-sulfur clusters. Therefore, regulated trafficking is required to prevent cell damages (Stohs and Bagchi, 1995; Macomber and Imlay, 2009; Kaplan and Maryon, 2016). Fluorescent copper indicators enable monitoring of free Cu^+^ concentration, thus leading to a better understanding of Cu^+^ dynamics and localizations under stimuli or stress. To the best of our knowledge, there are no fluorescent indicators designed to be specific for Cu^2+^.

### 9.2 Small Molecule-Based Cu^+^ Indicators

CTAP-1 is the first small molecule-based fluorescent indicator for Cu^+^ with a ∆*F*/*F*_0_ of 4.6 (Yang et al., 2005). The indicator consists of a tetrathiaza crown ether Cu^+^-binding motif and a pyrazoline fluorophore. The tetrathiaza crown ether allows specific binding to Cu^+^ (*K*_d_ = 40 nM) over other ions including Cu^2+^, and monovalent ions K^+^ and Na^+^. The pyrazoline fluorophore has a peak emission wavelength at 480 nm with UV excitation. CTAP-1 is membrane permeable and allows monitoring of Cu^+^ localization in NIH 3T3 cells grown in a medium supplemented with Cu^+^. One drawback of CTAP-1 is that it aggregates in cells. This issue was largely addressed with CTAP-2, which also exhibits an improved ∆*F*/*F*_0_ of 65 (Morgan et al., 2011).

Coppersensor-1 (CS1) is another small molecule-based Cu^+^ indicator based on BODIPY fluorophore and an azatetrathia binding domain (Zeng et al., 2006). The indicator displays a ∆*F*/*F*_0_ of 10 and peak excitation and emission wavelengths at 540 and 566 nm, respectively. CS3 is a more sensitive and brighter version with a ∆*F*/*F*_0_ of 75 (Dodani et al., 2011a, 20), enabling visualization of intracellular Cu^+^ concentration at basal and depleted levels and identification of Ca^2+^-dependent Cu^+^ redistribution.

Some small molecule-based Cu^+^ indicators were developed for more specific purposes. Mito-CS1 can be targeted to mitochondria *via* a triphenylphosphonium moiety and therefore enables Cu^+^ imaging in mitochondria (Dodani et al., 2011b). RCS1, a ratiometric indicator, has a ∆*R*/*R*_0_ of 20 and peak emission wavelengths at 505 and 570 nm *in vitro* and is effective as demonstrated in live C6 rat glioma cells (Domaille et al., 2010). ACu1 can be excited by two-photon at 750 nm and was used to image Cu^+^ in hippocampal slices at depths ranging from 90 to 220 µm (Su Lim et al., 2011). Cao Cu-3 is based on a tricarbocyanine scaffold and exhibits a ∆*F*/*F*_0_ of 9.6 with near-infrared fluorescence and was applied in live MG63 cells (Cao et al., 2012).

### 9.3 Genetically Encodable Cu^+^ Indicators

The apparent Cu^+^ affinities of the small molecule-based Cu^+^ indicators range from 10^−8^ to 10^−14^ M, which is typically too weak for the basal concentration of Cu^+^ in the cell. Genetically encodable Cu^+^ indicators address this issue with improved Cu^+^ affinities.

FRET-based genetically encodable Cu^+^ indicators have been developed using several Cu^+^-binding proteins. Wegner and coworkers reported Ace1-FRET, Mac1-FRET, and Amt1-FRET (Wegner et al., 2010, 2011). Amt1-FRET is based on a Cu^+^-responsive transcriptional regulator Amt1 and the CFP/YFP FRET pair (Wegner et al., 2010). The indicator has a high affinity (*K*_d_ = 2.5 × 10^–18^ M) that enables visualization of the tightly regulated Cu^+^. Ace1-FRET and Mac1-FRET are based on yeast copper regulators Ace1 and Mac1, respectively (Wegner et al., 2011). The two indicators show opposing responses to Cu^+^: Mac1-FRET decreases in FRET efficiency upon Cu^+^ binding and Ace1-FRET increases in FRET efficiency upon Cu^+^ binding. Ace1-FRET has a *K*_d_ of 4.7 × 10^–18^ ± 8.8 × 10^–19^ M and Mac1-FRET has a *K*_d_ of 9.7 × 10^–20^ ± 1.3 × 10^–20^ M for Cu^+^. The Zn^2+^ indicator eCALWY was also modified for Cu^+^ binding (Vinkenborg et al., 2009; Koay et al., 2013). The Zn^2+^-binding domains used in eCALWY are Cu^+^ chaperones ATOX1 and WD4, which contains CXXC Zn^2+^-binding motifs (Vinkenborg et al., 2009). Mutating several cysteines to methionines in the CXXC motifs abolished Zn^2+^ binding while retaining Cu^+^ binding (Koay et al., 2013). The resulting eCALWY-C2M/C3M shows specific binding to Cu^+^ (*K*_d_ = 10^−15^–10^−16^ M) over Zn^2+^ (*K*_d_ = 1.4 × 10^−6^ M) and no binding to Co^2+^, Cu^2+^, or Ni^2+^.

The single FP-based Cu^+^ indicators include EGFP-145Amt1, YFP-Ace1 and YAGn series. EGFP-145Amt1is based on Amt1 and ncpEGFP (Liang et al., 2012). YFP-Ace1 is based on Ace1 and YFP (Liu et al., 2013). YFP-Ace1 was optimized by adding GGS linkers of different lengths before N146 of YFP. The resulting variants were named YAGn where n denotes the number of GGS repeats in the linker. YFP-Ace1 and YAG1 are excitation ratiometric at 440 and 496 nm. The longer linkers abolish the ratiometric change. The fluorescent response is 38, 35, 30, 25, and 25% for YFP-Ace1, YAG1, YAG2, YAG3, and YAG4, respectively. The Cu^+^ affinity is increased with increasing linker lengths [*K*_d_ values for YFP-Ace1, YAG1, YAG2, YAG3, and YAG4 are (8.2 ± 1.2) × 10^−18^ M, (2.0 ± 0.8) × 10^−18^ M, (1.2 ± 1.0) × 10^−18^ M, (4.6 ± 1.2) × 10^−19^ M, and (3.3 ± 0.9) × 10^−19^ M].

## 10 Indicators For Toxic Ions (Pb^2+^, Cd^2+^, As^3+^ and Hg^2+^)

Indicators for toxic ions, Pb^2+^, Cd^2+^, As^3+^ and Hg^2+^ were developed for the general purpose of further investigating the mechanisms of their toxicities in cells. Many of these toxic ions can hijack the transporters for essential ions and thus disrupt their normal functions. Pd^2+^ can be imported through Ca^2+^ channels and N-methyl-D-aspartate (NMDA) receptor (Kerper and Hinkle, 1997; Mazzolini et al., 2001). It interferes with iron incorporation of hemoglobin and acts as a Ca^2+^ or Zn^2+^analogue to disrupt Ca^2+^-dependent signaling pathway and Zn^2+^-dependent activities (Martinez-Finley et al., 2012). Similarly, Cd^2+^ mimics Ca^2+^ and Zn^2+^, leading to detrimental effects on multiple organs (Bridges and Zalups, 2005). Mercury and mercury compounds are extremely toxic due to their reactivity with the thiol groups of cysteines and thus causes a detrimental effect on a variety of key enzymes (Clarkson and Magos, 2006). Arsenic can react with reduced thiol groups to disrupt protein metabolism in general; arsenic in the forms of arsenite and arsenate are particularly toxic to the nervous system and cardiovascular system by disrupting voltage-gated K^+^ channels (Medda et al., 2020). Small molecule-based and genetically encodable fluorescent indicators are thus valuable tools to investigate the toxicity of these elements. Among these indicators, small molecule-based fluorescent indicators for Pb^2+^, Cd^2+^, and Hg^2+^ have been previously reviewed by Kim and coworkers (Ha et al., 2012).

Genetically encodable arsenic indicators include SenALiBs and GEARs. SenALiB is based on an *E. coli* arsenic sensing domain ArsR flanked by CFP and YFP (Soleja et al., 2019). Further optimized variant SenALiB-676n has a higher affinity for As^3+^ (*K*_d_ = 0.676 × 10^–6^ M) compared to SenALiB (*K*_d_ = 2.6 × 10^–5^ M). The indicator exhibits a maximum 10% increase in FRET ratio. GEAR-CV1 is based on a bacterial As^3+^ responsive transcriptional factor AfArsR from *Acidithiobacillus ferrooxidans* and exhibits a Δ*R*/*R* of 15.8 ± 0.2% and a *K*_d_ of 84.9 µM (Khan et al., 2021). After further improvement, GEAR-CV2 exhibits a larger FRET ratio change of 22 ± 3.5%. The single GFP-based GEAR-G1 exhibits a Δ*F*/*F*_0_ of 31.6% upon As^3+^ binding.

Pb^2+^ indicators include leadfluor-1 (LF1), leadglow, and the protein-based Met-lead 1.59 (Vijverberg and Westerink, 2012). LF1 combines a fluorescein-like scaffold and a dicarboxylate pseudocrown binding moiety (He et al., 2006). The indicator is sensitive with a ∆*F*/*F*_0_ of 18 and specific to Pb^2+^ over Li^+^, Na^+^, K^+^, Mg^2+^, Ca^2+^, Mn^2+^, Fe^2+^, Co^2+^, Ni^2+^, Cu^2+^, Zn^2+^, Cd^2+^, and Hg^2+^. Leadglow is another sensitive and specific small molecule-based Pb^2+^ indicator (Marbella et al., 2009). The practicality of these two indicators is limited by their affinities for Pb^2+^ (LF1: *K*_d_ = 23 ± 4 μM; Leadglow: *K*_d_ = 217 nM) that are too low for detecting typical Pb^2+^ concentration in the environment [a limit of 72 nM Pb^2+^ in drinking water has been set by the United States Environmental Protection Agency (Santucci and Scully, 2020)]. The genetically encodable Met-lead 1.59 provides an alternative for detection of low Pb^2+^ concentrations. It is based on *Cupriavidus metallidurans* CH34 Pb^2+^-binding protein PbrR flanked by CFP and YFP, and exhibits an emission ratio (YFP/CFP) in the range of 3.3–5.7 and a biphasic response with *K*_d_ values of 69 nM and 22 μM (Chiu and Yang, 2012).

Cd^2+^ indicators include biologically compatible small molecule-based Liu Cd-1, Peng Cd-1, and Cheng Cd-1, as well as genetically encodable Cd-FRET. Liu Cd-1 consists of fluorescein and a thiosemicarbazide, and responds to Cd^2+^ with a Δ*F*/*F*_0_ of 1.5 (Liu et al., 2007). Peng Cd-1 is based on BODIPY fluorophore and presented ratiometric emission (655 nm in a Cd^2+^ free environment and 597 nm in a Cd^2+^ rich environment) (Peng et al., 2007). Cheng Cd-1 is another BODIPY-based Pb^2+^ indicator with superior sensitivity and affinity (Cheng et al., 2008). The fluorescence intensity of Cheng Cd-1 is enhanced by a maximum 195-fold with linear detection of Cd^2+^ in the nanomolar range. Cd-FRET was created by modifying Zn^2+^ indicator ZinCh-9 with a new (Cys)_4_ metal-binding motif at the interface of the FPs (Vinkenborg et al., 2011). Met-cad 1.57 is another genetically encodable FRET-based Cd^2+^ indicator with a Cd^2+^-binding protein CadR flanked by CFP and YFP (Chiu et al., 2013).

Various Hg^2+^ indicators have been developed to detect Hg^2+^. Among these indicators, Zhao Hg-1 and Lin Hg-1 are biologically compatible and demonstrate remarkable dynamic ranges (Δ*F*/*F*_0_) of 1,200 and 1,000, respectively, (Lin et al., 2010; Zhao et al., 2010). Rhodamine spirolactam-based Zhao Hg-1 is nonfluorescent unless bound to Hg^2+^, which converts the spirolactam to a ring-open amide form reversibly (Zhao et al., 2010). Lin Hg-1 is similarly based on thiolspirolactam to achieve high sensitivity (Lin et al., 2010). Zhao Hg-1 and Lin Hg-1 bind Hg^2+^ with *K*_d_ values of 4.6 × 10^−7^ M and 2.5 × 10^−5^ M, respectively. Compared to these indicators, BODIPY-based 8H-BDP and fluorescein-based Mercuryfluor-1 (MF1) bind Hg^2+^ with higher affinities (8H-BDP: *K*_d_ = 6.3 × 10^−19^ M; MF1: *K*_d_ = 7.0 × 10^−11^ M) but decreased fluorogenicity (8H-BDP: Δ*F*/*F*_0_ = 27; MF1: Δ*F*/*F*_0_ = 170) (Yoon et al., 2005; Lu et al., 2009). MF1 was used to detect Hg^2+^ levels in fish samples (Yoon et al., 2005). A Hg^2+^-induced fluorescence change was detected in HeLa cells using 8H-BDP (Lu et al., 2009).

## 11 Discussion

The decades of work on monatomic ion indicators have yielded many useful tools with diverse spectral properties, sensitivity, specificity, affinities, and brightness. In general, the most useful indicators have the following properties:

1) A large dynamic range that allows signal change to be easily detected.
2) High brightness to enable signal detection with a low concentration of the indicator.
3) A suitable affinity that is compatible with the ligand concentration in the target environment.
4) Fast kinetics to enable the detection of transient changes.
5) Specific targeting that allows measurement in specific cell types or subcellular compartments.
6) Colour variety that enables multiplexed and quantitative measurement.

Steps for making a successful indicator include **1)** finding an appropriate ligand-binding moiety and a fluorescent reporter, **2)** exploring different indicator designs, and finally **3)** extensive optimizations of spectral properties, response, affinity, kinetics, and other relevant properties. The properties of the ligand-binding moieties and the reporters can be assessed before initiating the development of an indicator, with the safe assumption that the resulting indicator will inherit some or all of these properties. That is, the binding moieties provide the ligand-binding properties, such as the affinity and specificity, and the fluorescent reporters provide the spectral properties.

Improving existing indicators can likewise involve choosing an alternative ligand binder or reporter. The green fluorescent mNeonGreen, for example, has been used to replace EGFP in Ca^2+^ indicators (Subach et al., 2020; Zarowny et al., 2020) and also used as a single FP Cl^−^ indicator (Tutol et al., 2019a). The brighter mNeonGreen allows an accordingly increased maximum brightness for mNeonGreen-based indicators. The availability of newly discovered or engineered binding moieties can inspire the development of novel indicators. For example, the discovery of Kbp enabled the development of genetically encodable potassium indicators with a superior specificity for K^+^ over Na^+^ (Ashraf et al., 2016; Bischof et al., 2017; Shen et al., 2019).

The initial development of indicators typically requires multiple designs. The pH indicators SNARFs, for example, were developed with multiple derivatives that exhibit different spectral properties and p*K*_a_ values (Whitaker et al., 1991). Creating a new single FP-based indicator typically involves attempts with cp and ncp designs to increase the probability of obtaining a ligand-sensitive design. NIR-GECO, for example, only responds to Ca^2+^ with the ncp mIFP (Qian et al., 2019). The initial construct of genetically encodable indicators rarely responds with optimal performance and thus requires further optimizations. The best example is the continuing optimization of the GCaMP series over the last 2 decades. The optimizations include site saturations on identified important sites and directed evolution. The screenings of variants were performed in *E. coli* and later hippocampal neurons, ultimately leading to the state-of-the-art GECIs (Chen et al., 2013; Dana et al., 2019; Zhang et al., 2021).

An important factor for the future development of genetically encodable monatomic ion indicators is the discovery and exploitation of new binding proteins. A new binding protein can potentially lead to a new monatomic ion indicator with a more favorable affinity, specificity, or kinetics. For example, the current Na^+^ indicators are limited by both the lack of variety (there is not a genetically encodable Na^+^ indicator available yet) and the lack of specificity for Na^+^ over K^+^. A new Na^+^-binding motif could potentially fill the void and provide practical tools for Na^+^ imaging. Such a development could be impactful, particularly if the resulting indicator could be used in combination with indicators for Ca^2+^ or K^+^.

In this review, we discussed small molecule-based and genetically encodable indicators for monatomic ions of biological importance. Beyond the scope of this review, chemigenetic indicators are an emerging group of tools where the advantages of small molecule-based dyes and genetically encodable tags complement each other. For example, such indicators could benefit from both the superior brightness and photostability of synthetic small molecules as well as the genetically-targetable expression of proteins (Abdelfattah et al., 2019; Deo et al., 2021).

The diverse collection of monatomic ion indicators has enabled discoveries in biology and medicine. With an ever-expanding toolset, the future of biomedical research will benefit from a vast library of highly specific and reliable indicators. At the same time, new technologies and approaches for designs and optimizations emerge to benefit tool developers who are creating more robust and practical monatomic ion indicators at a faster-than-ever pace.

## Abbreviations

BFP, blue fluorescent protein; CaM, calmodulin; CFP, cyan fluorescent protein; cp, circularly permuted; EC, extinction coefficient; FP, fluorescent protein; FRET, Förster resonance energy transfer; GECI, genetically encodable Ca^2+^ indicator; GEKI, genetically encodable K^+^ indicator; GEMI, genetically encodable Mg^2+^ indicator; GEZI, genetically encodable Zn^2+^ indicator; GFP, green fluorescent protein; ncp, non-circularly permuted; QY, quantum yield; RFP, red fluorescent protein; YFP, yellow fluorescent protein.
