# Supplementary material for: Fluorescent Indicators For Biological Imaging of Monatomic Ions
Source: Front Cell Dev Biol. 2022 Apr 27;10:885440. doi: 10.3389/fcell.2022.885440 (PMC9093666; doi:10.3389/fcell.2022.885440)
Supplement: Supplementary file 1 [file DataSheet1.docx]

Supplementary Table S1. Spectral and biophysical properties of selected GECIs.

The parameters listed are peak absorbance wavelength (λ_abs_), extinction coefficient (EC), peak emission wavelength (λ_em_), quantum yield (QY), brightness (the product of EC and QY), dissociation constant (*K*_d_), Hill coefficient (n_H_), *in vitro* Ca^2+^-dependent responses (∆*R*/*R*_0_ for ratiometric-pericam, GEM-GECO1, GEX-GECO1, and REX-GECO1; ∆*F*/*F*_0_ for others; negative numbers indicate inverse indicators), fluorescence response for a single action potential, and apparent acid dissociation constant (p*K*_a_). Some parameters are provided for both the Ca^2+^-free and Ca^2+^-bound states. NR, not reported.

| name | Ca^2+^ | λ_abs_ (nm) | EC (M^-1^ cm^-1^) | λ_em_ (nm) | QY | Brightness (mM^-1^cm^-1^) | *K*_d_ (nM) | n_H_ | *in vitro* ∆*F*/*F*_0_ or ∆*R*/*R*_0_^[[1]](#footnote-1)^ | ∆*F*/*F*_0_ for 1 AP | p*K*_a_ | References |
| --- | --- | --- | --- | --- | --- | --- | --- | --- | --- | --- | --- | --- |
| Camgaroo | - | 400/490 | NR | ~520 | NR | NR | 7000 | 1.6 | 6 | NR | 10.1 | (Baird et al., 1999, 199) |
|  | + |  | NR |  | NR | NR |  |  |  |  | 8.9 |  |
| Flash-pericam | - | 403 | 26800 | 514 | 0.04 | 1.07 | 700 | 0.7 | 7 | NR | NR | (Nagai et al., 2001) |
|  |  | 488 | 6300 | NR | NR | NR |  |  |  |  |  |  |
|  | + | 410 | 21200 | 514 | 0.2 | 4.24 |  |  |  |  | NR |  |
|  |  | 494 | 16900 | NR | NR | NR |  |  |  |  |  |  |
| Ratiometric-pericam | - | 418 | 24100 | 511 | 0.3 | 7.23 | 1700 | 1.1 | 9 | NR | NR | (Nagai et al., 2001) |
|  |  | 494 | 4100 | NR | NR | NR |  |  |  |  |  |  |
|  | + | 415 | 20500 | 517 | 0.18 | 3.69 |  |  |  |  | NR |  |
|  |  | 494 | 10300 | NR | NR | NR |  |  |  |  |  |  |
| Inverse-pericam | - | 503 | 59000 | 515 | 0.64 | 37.76 | 200 | 1 | -0.49 | NR | NR | (Nagai et al., 2001) |
|  | + | 490 | 44000 | 513 | 0.44 | 19.36 |  |  |  |  | NR |  |
| G-CaMP | − | 409 | 1100 | NR | NR | NR | 235 | 3.3 | 3.5 | NR | 8.1 | (Nakai et al., 2001) |
|  |  | 488 | 570 | 510 | 0.03 | 0.02 |  |  |  |  |  |  |
|  | + | 410 | 690 | NR | NR | NR |  |  |  |  | 7.1 |  |
|  |  | 487 | 1400 | 510 | 0.05 | 0.07 |  |  |  |  |  |  |
| G-CaMP1.6 | − | 404 | 5800 | NR | NR | NR | 146 | 3.8 | 3.9 | NR | 8.8 | (Ohkura et al., 2005) |
|  |  | 489 | 1100 | 510 | 0.56 | 0.62 |  |  |  |  |  |  |
|  | + | 403 | 5200 | NR | NR | NR |  |  |  |  | 8.2 |  |
|  |  | 488 | 3800 | 509 | 0.79 | 3.00 |  |  |  |  |  |  |
| G-CaMP2 | − | 400 | 11100 | NR | NR | NR | 146 | NR | ~4 | NR | NR | (Tallini et al., 2006) |
|  |  | 491 | 5200 | 511 | 0.7 | 3.64 |  |  |  |  |  |  |
|  | + | 401 | 5800 | NR | NR | NR |  |  |  |  | NR |  |
|  |  | 487 | 19000 | 508 | 0.93 | 17.67 |  |  |  |  |  |  |
| GCaMP3 | - | 399 | 36000 | NR | NR | NR | 405 | 2.1 | 11.3 | 0.20 | 8.4 | (Tian et al., 2009; Dana et al., 2019) |
|  |  | 496 | 11000 | 513 | 0.2 | 2.20 |  |  |  |  |  |  |
|  | + | 399 | 20000 | NR | NR | NR |  |  |  |  | 7.0 |  |
|  |  | 496 | 50000 | 513 | 0.44 | 22.00 |  |  |  |  |  |  |
| GCaMP5A | - | NR | NR | NR | NR | NR | 307 | 2.7 | 16.4 | NR | 8.7 | (Akerboom et al., 2012) |
|  | + | 497 | 56100 | NR | 0.65 | 36.47 |  |  |  |  | 6.8 |  |
| GCaMP5D | - | NR | NR | NR | NR | NR | 730 | 2.5 | 21.0 | NR | 8.9 | (Akerboom et al., 2012) |
|  | + | 497 | 25300 | NR | 0.67 | 16.95 |  |  |  |  | 7.4 |  |
| GCaMP5G | - | NR | NR | NR | NR | NR | 460 | 2.5 | 31.7 | 0.37 | 9.1 | (Akerboom et al., 2012; Dana et al., 2019) |
|  | + | 497 | 49300 | NR | 0.67 | 33.03 |  |  |  |  | 7.0 |  |
| G-GECO1.2 | - | 402 | 37000 | NR | NR | NR | 1150 | 2.1 | 22.0 | NR | 10.4 | (Zhao et al., 2011) |
|  |  | 498 | 2000 | 513 | 0.25 | 0.50 |  |  |  |  |  |  |
|  | + | 402 | 22000 | NR | NR | NR |  |  |  |  | 7.2 |  |
|  |  | 498 | 33000 | 513 | 0.36 | 11.90 |  |  |  |  |  |  |
| GEM-GECO1 | - | 397 | 34000 | 511 | 0.31 | 10.20 | 340 | 2.9 | 109.0 | NR | 6.2 | (Zhao et al., 2011) |
|  | + | 390 | 36000 | 455 | 0.18 | 6.50 |  |  |  |  |  |  |
| GEX-GECO1 | - | 392 | 32000 | NR | NR | NR | 318 | 2.8 | 25.0 | 1.21 | 6.7 | (Zhao et al., 2011; Dana et al., 2019) |
|  |  | 482 | 0 | 512 | 0.21 | 6.70 |  |  |  |  |  |  |
|  | + | 390 | 32000 | 506 | 0.19 | 6.08 |  |  |  |  | 6.1 |  |
|  |  | 482 | 2500 | 506 | 0.39 | 0.98 |  |  |  |  |  |  |
| B-GECO0.1 | - | 378 | 22000 | 446 | 0.02 | 0.44 | 164 | 2.6 | 6.0 | NR | 5.0 | (Zhao et al., 2011) |
|  | + | 378 | 23000 | 446 | 0.18 | 4.10 |  |  |  |  | 5.6 |  |
| GCaMP6s | - | 505 | 2910 | NR | NR | NR | 144 | 2.9 | 62.2 | 0.23 | 9.8 | (Chen et al., 2013) |
|  | + | 497 | 68500 | 515 | 0.61 | 41.79 |  |  |  |  | 6.2 |  |
| GCaMP6m | - | 505 | 20700 | NR | NR | NR | 167 | 3.0 | 37.1 | 0.13 | 8.7 | (Chen et al., 2013) |
|  | + | 497 | 38200 | 515 | 0.61 | 23.30 |  |  |  |  | 6.9 |  |
| GCaMP6f | - | 505 | 2800 | NR | NR | 1.65 | 375 | 2.3 | 50.8 | 0.19 | 8.8 | (Chen et al., 2013) |
|  | + | 497 | 61900 | 515 | 0.59 | 36.52 |  |  |  |  | 6.3 |  |
| jGCaMP7s | - | NR | 5554 | NR | 0.58 | 3.22 | 68 | 2.5 | 39.4 | 0.657 | 7.7 | (Dana et al., 2019) |
|  | + | NR | 70117 | NR | 0.65 | 45.58 |  |  |  |  | 6.4 |  |
| jGCaMP7f | - | NR | 2358 | NR | 0.47 | 1.11 | 174 | 2.3 | 29.2 | 0.316 | 7.9 | (Dana et al., 2019) |
|  | + | NR | 56028 | NR | 0.59 | 33.06 |  |  |  |  | 6.5 |  |
| jGCaMP7b | - | NR | 5668 | NR | 0.59 | 3.34 | 82 | 3.1 | 21.1 | 0.406 | 7.8 | (Dana et al., 2019) |
|  | + | NR | 56562 | NR | 0.6 | 33.94 |  |  |  |  | 6.4 |  |
| jGCaMP7c | - | NR | 1541 | NR | 0.5 | 0.77 | 298 | 2.4 | 144.6 | 0.223 | 8.7 | (Dana et al., 2019) |
|  | + | NR | 49566 | NR | 0.59 | 29.24 |  |  |  |  | 6.7 |  |
| XCaMP-G | - | 398 | 50100 | 512 | 0.14 | 1.8 | 200 | 1.8 | 10.1 | 1.97 | 7.2 | (Inoue et al., 2019; Zhang et al., 2021) |
|  |  | 487 | 13200 |  |  |  |  |  |  |  |  |  |
|  | + | 399 | 36600 | 514 | 0.39 | 19.9 |  |  |  |  | 8.4 |  |
|  |  | 487 | 51400 |  |  |  |  |  |  |  |  |  |
| XCaMP-Gf | - | 401 | 97280 | 514 | 0.18 | 0.9 | 115 | 1.4 | 11.7 | 2.05 | 6.1 | (Inoue et al., 2019; Zhang et al., 2021) |
|  |  | 492 | 5220 |  |  |  |  |  |  |  |  |  |
|  | + | 394 | 82390 | 514 | 0.5 | 11.4 |  |  |  |  | 8.7 |  |
|  |  | 492 | 22530 |  |  |  |  |  |  |  |  |  |
| XCaMP-Y | - | 418 | 47300 | 525 | 0.11 | 1.7 | 81 | 1.5 | 6.2 | NR | 7.5 | (Inoue et al., 2019) |
|  |  | 498 | 16000 |  |  |  |  |  |  |  |  |  |
|  | + | 415 | 34500 | 527 | 0.26 | 12.3 |  |  |  |  | 9.4 |  |
|  |  | 503 | 47400 |  |  |  |  |  |  |  |  |  |
| jGCaMP8s | - | 496 | 2120 | 513 | NR | NR | 46 | 2.2 | 49.5 | NR | 7.7 | (Zhang et al., 2021) |
|  | + |  | 57000 |  | NR | NR |  |  |  |  | 6.5 |  |
| jGCaMP8f | - | 496 | 1930 | 512 | NR | NR | 334 | 2.1 | 78.8 | 3.1 | 7.7 | (Zhang et al., 2021) |
|  | + |  | 51000 |  | NR | NR |  |  |  |  | 6.7 |  |
| jGCaMP8m | - | 496 | 2250 | 512 | NR | NR | 108 | 1.9 | 45.7 | 5.0 | 7.4 | (Zhang et al., 2021) |
|  | + |  | 50000 |  | NR | NR |  |  |  |  | 6.7 |  |
| mNG-GECO1 | + | 496 | 102000 | 513 | 0.69 | 70.38 | 807 | NR | 44 | NR | NR | (Zarowny et al., 2020) |
| NCaMP7 | - | 402/406 | 46600 | 520 | 0.048 | 2.2368 | 96 | 2.2 | 88 | NR | 5.4/6.6 | (Subach et al., 2020) |
|  | + | 509/512 | 110000 | 522 | 0.52 | 57.2 | NR |  |  |  | 6.18 |  |
| R-GECO1 | - | 445 | 22000 | NR | NR | NR | 482 | 2.06 | 15.0 | NR | 8.9 | (Zhao et al., 2011) |
|  |  | 577 | 15000 | 600 | 0.06 | 0.72 |  |  |  |  |  |  |
|  | + | 445 | 9000 | NR | NR | NR |  |  |  |  | 6.6 |  |
|  |  | 561 | 51000 | 589 | 0.2 | 10.20 |  |  |  |  |  |  |
| R-GECO1.2 | - | 564 | 2800 | 595 | 0.16 | 0.45 | 1200 | 2.79 | 32.0 | NR | 8.9 | (Wu et al., 2013) |
|  | + | 556 | 52000 | 585 | 0.29 | 15.10 |  |  |  |  | 6.0 |  |
| O-GECO1 | - | 545 | 1400 | 570 | 0.07 | 0.10 | 1500 | 2.06 | 145.0 | NR | 9.4 | (Wu et al., 2013) |
|  | + | 543 | 65000 | 564 | 0.22 | 14.30 |  |  |  |  | 6.1 |  |
| CAR-GECO1 | - | 565 | 2500 | 620 | 0.11 | 0.28 | 490 | 2.01 | 26.0 | NR | 9.1 | (Wu et al., 2013) |
|  | + | 560 | 36000 | 609 | 0.21 | 7.60 |  |  |  |  | 5.7 |  |
| REX-GECO1 | - | 582 | 26000 | 600 | 0.06 | 1.60 | 240 | 1.8 | 99.0 | NR | 6.5 | (Wu et al., 2014) |
|  | + | 480 | 28000 | 585 | 0.23 | 6.40 |  |  |  |  |  |  |
| RCaMP1f | - | 574 | 17400 | 597 | 0.11 | 1.91 | 1900 | 2.80 | 11.3 | NR | 4.6 | (Akerboom et al., 2013) |
|  | + | 572 | 58900 | 592 | 0.48 | 28.27 |  |  |  |  | 5.3 |  |
| RCaMP1h | - | 575 | 18700 | 602 | 0.14 | 2.62 | 1300 | 2.50 | 9.0 | NR | 3.2/4.7/6.5 | (Akerboom et al., 2013) |
|  | + | 571 | 65100 | 594 | 0.51 | 33.20 |  |  |  |  | 4.9 |  |
| RCaMP2 | - | 445 | 28288 | NR | NR | NR | 69 | 1.2 | 3.8 | 0.09 | 8.7 | (Inoue et al., 2015) |
|  |  | 576 | 14200 | 591 | 0.11 | 1.59 |  |  |  |  |  |  |
|  | + | 445 | 16600 | NR | NR | NR |  |  |  |  | 6.1 |  |
|  |  | 563 | 48100 | 583 | 0.23 | 11.2 |  |  |  |  |  |  |
| jRCaMP1a | - | ~575 | 33800 | ~600 | NR | NR | 214 | 0.86 | 2.2 | 0.15 | 5.6 | (Dana et al., 2016) |
|  | + | ~570 | 54100 | ~595 | NR | NR |  |  |  |  | 6.4 |  |
| jRCaMP1b | - | ~575 | 25300 | ~600 | NR | NR | 712 | 1.6 | 6.2 | 0.09 | 6.4 | (Dana et al., 2016) |
|  | + | ~570 | 53400 | ~595 | NR | NR |  |  |  |  | 5.5 |  |
| jRGECO1a | - | ~580 | 61800 | ~600 | NR | NR | 148 | 1.9 | 10.6 | 0.3 | 8.6 | (Dana et al., 2016) |
|  | + | ~570 | 53300 | ~590 | NR | NR |  |  |  |  | 6.3 |  |
| XCaMP-R | - | 444 | 51600 | 598 | 0.14 | 3.3 | 97 | 1.1 | 5.6 | NR | 6.1 | (Inoue et al., 2019) |
|  |  | 574 | 37700 |  |  |  |  |  |  |  |  |  |
|  | + | 452 | 37900 | 593 | 0.28 | 21.7 |  |  |  |  | 8.7 |  |
|  |  | 561 | 76300 |  |  |  |  |  |  |  |  |  |
| K-GECO1 | - | 568 | 19000 | 594 | 0.12 | 2.28 | 165 | 1.12 | 11 | NR | NR | (Shen et al., 2018) |
|  | + | 565 | 61000 | 590 | 0.45 | 27.45 |  |  |  |  | NR |  |
| NIR-GECO1 | - | 678 | 62000 | 704 | 0.063 | 3.906 | 885 | 1.03 | -0.90 | -0.045 | 6.03 | (Qian et al., 2019) |
|  | + | 678 | 20000 | 704 | 0.019 | 0.38 |  |  |  |  | 4.68 |  |
| NIR-GECO2 | - | NR | 67000 | NR | 0.059 | 3.953 | 331 | 0.94 | -0.94 | -0.16 | 5.26 | (Qian et al., 2020) |
|  | + | NR | 18000 | NR | 0.014 | 0.252 |  |  |  |  | 4.85 |  |
| NIR-GECO2G | - | NR | 74000 | NR | 0.061 | 4.514 | 480 | 0.78 | -0.90 | -0.17 | 5.34 | (Qian et al., 2020) |
|  | + | NR | 21000 | NR | 0.021 | 0.441 |  |  |  |  | 4.84 |  |

Supplementary Table S2. Physical properties of selected small molecule-based Mg^2+^ indicators.

The parameters listed are peak absorbance wavelength (λ_abs_), extinction coefficient (EC), peak emission wavelength (λ_em_), and apparent dissociation constant (*K*_d_) for Mg^2+^ and Ca^2+^. Some parameters were provided at both Mg^2+^-free and Mg^2+^-bound states.

| name | Mg^2+^ | λ_abs_ (nm) | EC (M^-1^cm^-1^) | λ_em_ (nm) | *K*_d_ for Mg^2+^ (mM) | *K*_d_ for Ca^2+^ (µM) | References |
| --- | --- | --- | --- | --- | --- | --- | --- |
| mag-fura-2 | - | 369 | 22,000 | 511 | 1.5 | 53 | (Raju et al., 1989, 1; Meuwis et al., 1998) |
|  | + | 330 | 24,000 | 491 |  |  |  |
| mag-fura-5 | - | 369 | 23,000 | 505 | 2.3 | 28 | (Illner et al., 1992; Claflin et al., 1994) |
|  | + | 332 | 25,000 | 482 |  |  |  |
| mag-indo-1 | - | 349 | 38,000 | 480 | 2.7 | 35 | (Csernoch et al., 1998) |
|  | + | 330 | 33,000 | 417 |  |  |  |
| mag-fluo-4 | - | 490 | 74,000 | NR | 4.7 | 22 | (Zhao et al., 1996; Lee et al., 2009) |
|  | + | 493 | 75,000 | 517 |  |  |  |
| Magnesium Green | - | 506 | 77,000 | 531 | 1.0 | 6 | (Zhao et al., 1996; Shmigol et al., 2001) |
|  | + | 506 | 75,000 | 531 |  |  |  |
|  | + | 578 | 82,000 | 603 |  |  |  |
| KMG-104-AsH | - | 520 | NR | 540 | 1.7 | 100 | (Fujii et al., 2014) |
|  | + | 521 | NR | 540 |  |  |  |

Supplementary Table S3. Key properties of selected small molecule-based and genetically encodable pH indicators

The parameters listed are peak absorbance wavelength (λ_abs_), extinction coefficient (EC), peak emission wavelength (λ_em_), quantum yield (QY), isosbestic wavelength (λ_iso_), dissociation constant (*K*_d_), Hill coefficient (n_H_), and *in vitro* fluorescent responses (∆*R*/*R*_0_ as (*R*_basic_-*R*_acidic_)/*R*_acidic_ for r-pHluorin, where *R* is calculated as *F*_475nm_/*F*_395nm_, or ∆*F*/*F*_0_ as (*F*_basic_-*F*_acidic_)/*F*_acidic_ for others). Some parameters are provided for both acidic and basic conditions.NR, not reported. NA, not applicable.

| name | pH | λ_abs_ (nm) | EC (M^-1^cm^-1^) | λ_em_ (nm) | QY | λ_iso_ (nm) | p*K*_a_ | n_H_ | ∆*F*/*F*_0_  or ∆*R*/*R*_0_^[[2]](#footnote-2)^ | References |
| --- | --- | --- | --- | --- | --- | --- | --- | --- | --- | --- |
| **Small molecule-based pH indicators** | | | | | | | | | | |
| BCECF | acidic | 470 | NR | NR | NR | 440 | 6.98 | NR | NR | (Rink et al., 1982) |
|  |  | 505 | NR | 535 | NR |  |  |  |  |  |
|  | basic | 470 | NR | NR | NR |  |  |  |  |  |
|  |  | 505 | NR | 520 | NR |  |  |  |  |  |
| SNARF-1 | acidic | 515 | 17700 | 583 | 0.03 | 521 | 7.62 | NR | NR | (Whitaker et al., 1991) |
|  |  | 544 | 21600 |  |  |  |  |  |  |  |
|  | basic | 573 | 44100 | 631 | 0.09 |  |  |  |  |  |
| SNARF-2 | acidic | 518 | 20500 | 584 | 0.03 | 530 | 7.43, 7.50 | NR | NR | (Whitaker et al., 1991) |
|  |  | 550 | 24400 |  |  |  |  |  |  |  |
|  | basic | 576 | 46400 | 633 | 0.16 |  |  |  |  |  |
| C.SNARF-1 | acidic | 518 | 23200 | 585 | 0.047 | 534 | 7.6 | NR | NR | (Whitaker et al., 1991) |
|  |  | 548 | 25700 |  |  |  |  |  |  |  |
|  | basic | 575 | 44900 | 637 | 0.09 |  |  |  |  |  |
| C.SNARF-2 | acidic | 516 | 26200 | 585 | 0.02 | 524 | 7.70, 7.78 | NR | NR | (Whitaker et al., 1991) |
|  |  | 550 | 29800 |  |  |  |  |  |  |  |
|  | basic | 577 | 51500 | 635 | 0.11 |  |  |  |  |  |
| **Genetically encodable pH indicators** | | | | | | | | | | |
| EGFP | NA | 480 | NR | 510 | NR | NR | 6.15 | 0.7 | NR | (Kneen et al., 1998) |
| EYFP | NA | 514 | NR | 527 | NR | NR | 7.1 | 1.1 | NR | (Llopis et al., 1998) |
| mNecterine | NA | 558 | NR | 578 | NR | NR | 6.9 | 0.78 | NR | (Johnson et al., 2009) |
| E^2^GFP | acidic | 424 | 31560 | 510 | 0.22 | NR | 7 | NR | NR | (Bizzarri et al., 2006) |
|  | basic | 401 | 28180 | 523 | 0.91 |  |  |  |  |  |
|  |  | 515 | 22400 | 527 | NR |  |  |  |  |  |
| dEGFP1 | acidic | 400 | 28700 | NR | NR | NR | 8.02 | NR | NR | (Hanson et al., 2002) |
|  | basic | 504 | 54800 | 516 | 0.49 |  |  |  |  |  |
| dEGFP2 | acidic | 398 | 21700 | NR | NR | NR | 7.25 | NR | NR | (Hanson et al., 2002) |
|  | basic | 496 | 38200 | 517 | 0.55 |  |  |  |  |  |
| dEGFP3 | acidic | 396 | 26700 | NR | NR | NR | 6.86 | NR | NR | (Hanson et al., 2002) |
|  | basic | 508 | 45900 | 518 | 0.57 |  |  |  |  |  |
| dEGFP4 | acidic | 400 | 26900 | NR | NR | NR | 7.37 | NR | NR | (Hanson et al., 2002) |
|  | basic | 509 | 50800 | 518 | 0.27 |  |  |  |  |  |
| r-pHluorin | NA | 395 | NR | 508 | NR | NR | NR | NR | -0.8 (pH 5.5–7.5) | (Miesenböck et al., 1998) |
|  |  | 475 | NR |  |  |  |  |  |  |  |
| e-pHluorin | NA | 395 | NR | 508 | NR | NR | NR | NR | 5 (pH 6–7.5) | (Miesenböck et al., 1998) |
| pHRed | NA | 440 | NR | 610 | NR | NR | 6.5 | NR | >9 | (Tantama et al., 2011) |
| pHTomato | NA | 550 | NR | 580 | NR | NR | 7.8 | NR | >2 (pH 7.5–9.8) | (Li and Tsien, 2012) |
| pHuji | NA | 556 | 31000 | 598 | 0.22 | NR | 7.7 | NR | 21 (pH 5.5–7.5) | (Shen et al., 2014; Liu et al., 2021) |
| SE-pHluorin | NA | 495 | 45000 | 512 | 0.52 | NR | 7.2 | 1.90 | 49 (pH 5.5–7.5) | (Sankaranarayanan et al., 2000; Shen et al., 2014; Liu et al., 2021) |
| pHoran1 | NA | 547 | NR | 564 | NR | NR | 6.7 | 0.87 | 9 (pH 5.5–7.5) | (Shen et al., 2014) |
| pHoran2 | NA | 549 | NR | 563 | NR | NR | 7.0 | 0.89 | 11 (pH 5.5–7.5) | (Shen et al., 2014) |
| pHoran3 | NA | 551 | NR | 566 | NR | NR | 7.4 | 0.87 | 14 (pH 5.5–7.5) | (Shen et al., 2014) |
| pHoran4 | NA | 547 | NR | 561 | NR | NR | 7.5 | 0.92 | 16 (pH 5.5–7.5) | (Shen et al., 2014) |
| pHmScarlet | NA | 562 | 85000 | 585 | 0.47 | NR | 7.4 | 1.1 | 26 (pH 5.5–7.5) | (Liu et al., 2021) |

Supplementary Table S4. Physical properties of genetically encodable Cl^-^ indicators.

The parameters listed are peak absorbance wavelength (λ_abs_), extinction coefficient (EC), peak emission wavelength (λ_em_), quantum yield (QY), brightness (the product of EC and QY), dissociation constant (*K*_d_), Hill coefficient (n_H_), *in vitro* dynamic range (∆*R*/*R*_0_ for Clomeleon and SuperClomeleon, and ∆*F*/*F*_0_ for others; negative sign indicates inverse indicators), and acid dissociation constant (p*K*_a_). Some parameters were provided at both Cl^-^-free and Cl^-^-rich states.

| name | Cl^-^ | λ_abs_ (nm) | EC (M^-1^cm^-1^) | λ_em_ (nm) | QY | brightness (mM^-1^cm^-1^) | *K*_d_ (mM) | n_H_ | ∆*F*/*F*_0_  or ∆*R*/*R*_0_^[[3]](#footnote-3)^ | p*K*_a_ | References |
| --- | --- | --- | --- | --- | --- | --- | --- | --- | --- | --- | --- |
| mNeonGreen | - | 400 | NR | NR | NR | NR | 9.8 | 0.72 | 20 | 5.7 | (Tutol et al., 2019a) |
|  |  | 505 | 10021 | 520 | 0.18 | 1.80 |  |  |  |  |  |
|  | + | 400 | NR | NR | NR | NR |  |  |  | 4.7 |  |
|  |  | 505 | 26884 | 520 | 0.4 | 10.75 |  |  |  |  |  |
| YFP | / | 514 | NR | 527 | NR | NR | 777 | NR | -0.4 | NR | (Wachter and James Remington, 1999) |
| Clomeleon | / | NR | NR | NR | NR | NR | 167 | NR | -0.8 | NR | (Kuner and Augustine, 2000, 200) |
| SuperClomeleon | / | NR | NR | NR | NR | NR | 8.1 | NR | -0.9 | NR | (Grimley et al., 2013) |
| YFP-H148Q | / | NR | 71000 | NR | 0.59 | NR | 100 | NR | -0.5 | 6.7 | (Jayaraman et al., 2000) |
| YFP-H148Q/I152L | / | NR | 52200 | NR | 0.6 | NR | 85 | NR | -0.5 | 6.92 | (Galietta et al., 2001) |
| phi-YFP | - | 480 | NR | 540 | 0.44 | NR | 384 | 0.9 | 2.5 | 4.9 | (Tutol et al., 2019b) |
|  |  | 400 | NR | 540 | 0.02 | NR |  |  |  |  |  |
|  | + | 480 | NR | 540 | 0.49 | NR |  |  |  | 5.4 |  |
|  |  | 400 | NR | 540 | 0.06 | NR |  |  |  |  |  |

**References**

Akerboom, J., Carreras Calderón, N., Tian, L., Wabnig, S., Prigge, M., Tolö, J., et al. (2013). Genetically Encoded Calcium Indicators for Multi-color Neural Activity Imaging and Combination with Optogenetics. *Front. Mol. Neurosci.* 6. doi:10.3389/fnmol.2013.00002.

Akerboom, J., Chen, T.-W., Wardill, T. J., Tian, L., Marvin, J. S., Mutlu, S., et al. (2012). Optimization of a GCaMP Calcium Indicator for Neural Activity Imaging. *J. Neurosci.* 32, 13819–13840. doi:10.1523/JNEUROSCI.2601-12.2012.

Baird, G. S., Zacharias, D. A., and Tsien, R. Y. (1999). Circular Permutation and Receptor Insertion within Green Fluorescent Proteins. *PNAS* 96, 11241–11246. doi:10.1073/pnas.96.20.11241.

Bizzarri, R., Arcangeli, C., Arosio, D., Ricci, F., Faraci, P., Cardarelli, F., et al. (2006). Development of a Novel GFP-based Ratiometric Excitation and Emission pH Indicator for Intracellular Studies. *Biophysical Journal* 90, 3300–3314. doi:10.1529/biophysj.105.074708.

Chen, T.-W., Wardill, T. J., Sun, Y., Pulver, S. R., Renninger, S. L., Baohan, A., et al. (2013). Ultrasensitive Fluorescent Proteins for Imaging Neuronal Activity. *Nature* 499, 295–300. doi:10.1038/nature12354.

Claflin, D. R., Morgan, D. L., Stephenson, D. G., and Julian, F. J. (1994). The Intracellular Ca^2+^ Transient and Tension in Frog Skeletal Muscle Fibres Measured with High Temporal Resolution. *J Physiol* 475, 319–325. doi:10.1113/jphysiol.1994.sp020072.

Csernoch, L., Bernengo, J. C., Szentesi, P., and Jacquemond, V. (1998). Measurements of Intracellular Mg^2+^ Concentration in Mouse Skeletal Muscle Fibers with the Fluorescent Indicator Mag-indo-1. *Biophys. J.* 75, 957–967. doi:10.1016/S0006-3495(98)77584-8.

Dana, H., Mohar, B., Sun, Y., Narayan, S., Gordus, A., Hasseman, J. P., et al. (2016). Sensitive Red Protein Calcium Indicators For Imaging Neural Activity. *Elife* 5. doi:10.7554/eLife.12727.

Dana, H., Sun, Y., Mohar, B., Hulse, B. K., Kerlin, A. M., Hasseman, J. P., et al. (2019). High-performance Calcium Sensors for Imaging Activity in Neuronal Populations and Microcompartments. *Nature Methods* 16, 649–657. doi:10.1038/s41592-019-0435-6.

Fujii, T., Shindo, Y., Hotta, K., Citterio, D., Nishiyama, S., Suzuki, K., et al. (2014). Design and Synthesis of a FlAsH-Type Mg^2+^ Fluorescent Probe for Specific Protein Labeling. *J. Am. Chem. Soc.* 136, 2374–2381. doi:10.1021/ja410031n.

Galietta, L. J. V., Haggie, P. M., and Verkman, A. S. (2001). Green Fluorescent Protein-Based Halide Indicators with Improved Chloride and Iodide Affinities. *FEBS Letters* 499, 220–224. doi:10.1016/S0014-5793(01)02561-3.

Grimley, J. S., Li, L., Wang, W., Wen, L., Beese, L. S., Hellinga, H. W., et al. (2013). Visualization of Synaptic Inhibition with an Optogenetic Sensor Developed by Cell-Free Protein Engineering Automation. *J. Neurosci.* 33, 16297–16309. doi:10.1523/JNEUROSCI.4616-11.2013.

Hanson, G. T., McAnaney, T. B., Park, E. S., Rendell, M. E. P., Yarbrough, D. K., Chu, S., et al. (2002). Green Fluorescent Protein Variants as Ratiometric Dual Emission pH Sensors. 1. Structural Characterization and Preliminary Application. *Biochemistry* 41, 15477–15488. doi:10.1021/bi026609p.

Illner, H., McGuigan, J. A. S., and Lüthi, D. (1992). Evaluation of Mag-fura-5, the New Fluorescent Indicator for Free Magnesium Measurements. *Pflügers Arch* 422, 179–184. doi:10.1007/BF00370418.

Inoue, M., Takeuchi, A., Horigane, S., Ohkura, M., Gengyo-Ando, K., Fujii, H., et al. (2015). Rational Design of a High-Affinity, Fast, Red Calcium Indicator R-CaMP2. *Nat. Methods* 12, 64–70. doi:10.1038/nmeth.3185.

Inoue, M., Takeuchi, A., Manita, S., Horigane, S., Sakamoto, M., Kawakami, R., et al. (2019). Rational Engineering of XCaMPs, a Multicolor GECI Suite for *In Vivo* Imaging of Complex Brain Circuit Dynamics. *Cell* 177, 1346-1360.e24. doi:10.1016/j.cell.2019.04.007.

Jayaraman, S., Haggie, P., Wachter, R. M., Remington, S. J., and Verkman, A. S. (2000). Mechanism and Cellular Applications of a Green Fluorescent Protein-Based Halide Sensor. *J. Biol. Chem.* 275, 6047–6050. doi:10.1074/jbc.275.9.6047.

Johnson, D. E., Ai, H.-W., Wong, P., Young, J. D., Campbell, R. E., and Casey, J. R. (2009). Red Fluorescent Protein pH Biosensor to Detect Concentrative Nucleoside Transport. *J. Biol. Chem.* 284, 20499–20511. doi:10.1074/jbc.M109.019042.

Kneen, M., Farinas, J., Li, Y., and Verkman, A. S. (1998). Green Fluorescent Protein as a Noninvasive Intracellular pH Indicator. *Biophys J* 74, 1591–1599.

Kuner, T., and Augustine, G. J. (2000). A Genetically Encoded Ratiometric Indicator for Chloride: Capturing Chloride Transients in Cultured Hippocampal Neurons. *Neuron* 27, 447–459. doi:10.1016/s0896-6273(00)00056-8.

Lee, S., Lee, H. G., and Kang, S. H. (2009). Real-Time Observations of Intracellular Mg^2+^ Signaling and Waves in a Single Living Ventricular Myocyte Cell. *Anal. Chem.* 81, 538–542. doi:10.1021/ac8013324.

Li, Y., and Tsien, R. W. (2012). pHTomato: A Genetically-Encoded Indicator That Enables Multiplex Interrogation of Synaptic Activity. *Nat Neurosci* 15, 1047–1053. doi:10.1038/nn.3126.

Liu, A., Huang, X., He, W., Xue, F., Yang, Y., Liu, J., et al. (2021). pHmScarlet Is a pH-Sensitive Red Fluorescent Protein to Monitor Exocytosis Docking and Fusion Steps. *Nat Commun* 12, 1413. doi:10.1038/s41467-021-21666-7.

Llopis, J., McCaffery, J. M., Miyawaki, A., Farquhar, M. G., and Tsien, R. Y. (1998). Measurement of Cytosolic, Mitochondrial, and Golgi pH in Single Living Cells with Green Fluorescent Proteins. *PNAS* 95, 6803–6808. doi:10.1073/pnas.95.12.6803.

Meuwis, K., Boens, N., Gallay, J., and Vincent, M. (1998). Photophysics of Mag-fura-2: A Fluorescent Indicator for Intracellular Mg^2+^. *Chemical Physics Letters* 287, 412–420. doi:10.1016/S0009-2614(98)00178-X.

Miesenböck, G., De Angelis, D. A., and Rothman, J. E. (1998). Visualizing Secretion and Synaptic Transmission with pH-Sensitive Green Fluorescent Proteins. *Nature* 394, 192–195. doi:10.1038/28190.

Nagai, T., Sawano, A., Park, E. S., and Miyawaki, A. (2001). Circularly Permuted Green Fluorescent Proteins Engineered to Sense Ca^2+^. *PNAS* 98, 3197–3202. doi:10.1073/pnas.051636098.

Nakai, J., Ohkura, M., and Imoto, K. (2001). A High Signal-To-Noise Ca^2+^ Probe Composed of a Single Green Fluorescent Protein. *Nature Biotechnology* 19, 137–141. doi:10.1038/84397.

Ohkura, M., Matsuzaki, M., Kasai, H., Imoto, K., and Nakai, J. (2005). Genetically Encoded Bright Ca^2+^ Probe Applicable for Dynamic Ca^2+^ Imaging of Dendritic Spines. *Anal. Chem.* 77, 5861–5869. doi:10.1021/ac0506837.

Qian, Y., Cosio, D. M. O., Piatkevich, K. D., Aufmkolk, S., Su, W.-C., Celiker, O. T., et al. (2020). Improved Genetically Encoded Near-Infrared Fluorescent Calcium Ion Indicators for *in Vivo* Imaging. *PLOS Biology* 18, e3000965. doi:10.1371/journal.pbio.3000965.

Qian, Y., Piatkevich, K. D., McLarney, B., Abdelfattah, A. S., Mehta, S., Murdock, M. H., et al. (2019). A Genetically Encoded Near-Infrared Fluorescent Calcium Ion Indicator. *Nat Methods* 16, 171–174. doi:10.1038/s41592-018-0294-6.

Raju, B., Murphy, E., Levy, L. A., Hall, R. D., and London, R. E. (1989). A Fluorescent Indicator for Measuring Cytosolic Free Magnesium. *Am. J. Physiol. Cell Physiol.* 256, C540–C548. doi:10.1152/ajpcell.1989.256.3.C540.

Rink, T. J., Tsien, R. Y., and Pozzan, T. (1982). Cytoplasmic pH and Free Mg^2+^ in Lymphocytes. *J. Cell Biol.* 95, 189–196. doi:10.1083/jcb.95.1.189.

Sankaranarayanan, S., De Angelis, D., Rothman, J. E., and Ryan, T. A. (2000). The Use of pHluorins for Optical Measurements of Presynaptic Activity. *Biophys. J.* 79, 2199–2208. doi:10.1016/S0006-3495(00)76468-X.

Shen, Y., Dana, H., Abdelfattah, A. S., Patel, R., Shea, J., Molina, R. S., et al. (2018). A Genetically Encoded Ca^2+^ Indicator Based on Circularly Permutated Sea Anemone Red Fluorescent Protein eqFP578. *BMC Biol.* 16, 9. doi:10.1186/s12915-018-0480-0.

Shen, Y., Rosendale, M., Campbell, R. E., and Perrais, D. (2014). pHuji, a PH-Sensitive Red Fluorescent Protein for Imaging of Exo- And Endocytosis. *J. Cell Biol.* 207, 419–432. doi:10.1083/jcb.201404107.

Shmigol, A. V., Eisner, D. A., and Wray, S. (2001). Simultaneous Measurements of Changes in Sarcoplasmic Reticulum and Cytosolic [Ca^2+^] in Rat Uterine Smooth Muscle Cells. *J. Physiol.* 531, 707–713. doi:10.1111/j.1469-7793.2001.0707h.x.

Subach, O. M., Sotskov, V. P., Plusnin, V. V., Gruzdeva, A. M., Barykina, N. V., Ivashkina, O. I., et al. (2020). Novel Genetically Encoded Bright Positive Calcium Indicator NCaMP7 Based on the mNeonGreen Fluorescent Protein. *Int J Mol Sci* 21. doi:10.3390/ijms21051644.

Tallini, Y. N., Ohkura, M., Choi, B.-R., Ji, G., Imoto, K., Doran, R., et al. (2006). Imaging Cellular Signals in the Heart *in Vivo*: Cardiac Expression of the High-Signal Ca^2+^ Indicator GCaMP2. *PNAS* 103, 4753–4758. doi:10.1073/pnas.0509378103.

Tantama, M., Hung, Y. P., and Yellen, G. (2011). Imaging Intracellular pH in Live Cells with a Genetically Encoded Red Fluorescent Protein Sensor. *J. Am. Chem. Soc.* 133, 10034–10037. doi:10.1021/ja202902d.

Tian, L., Hires, S. A., Mao, T., Huber, D., Chiappe, M. E., Chalasani, S. H., et al. (2009). Imaging Neural Activity in Worms, Flies and Mice with Improved GCaMP Calcium Indicators. *Nat. Methods* 6, 875–881. doi:10.1038/nmeth.1398.

Tutol, J. N., Kam, H. C., and Dodani, S. C. (2019a). Identification of mNeonGreen as a pH-Dependent, Turn-On Fluorescent Protein Sensor for Chloride. *ChemBioChem* 20, 1759–1765. doi:10.1002/cbic.201900147.

Tutol, J. N., Peng, W., and Dodani, S. C. (2019b). Discovery and Characterization of a Naturally Occurring, Turn-On Yellow Fluorescent Protein Sensor for Chloride. *Biochemistry* 58, 31–35. doi:10.1021/acs.biochem.8b00928.

Wachter, R. M., and James Remington, S. (1999). Sensitivity of the Yellow Variant of Green Fluorescent Protein to Halides and Nitrate. *Curr. Biol.* 9, R628–R629. doi:10.1016/S0960-9822(99)80408-4.

Whitaker, J. E., Haugland, R. P., and Prendergast, F. G. (1991). Spectral and Photophysical Studies of Benzo[c]xanthene Dyes: Dual Emission pH Sensors. *Anal. Biochem.* 194, 330–344. doi:10.1016/0003-2697(91)90237-n.

Wu, J., Abdelfattah, A. S., Miraucourt, L. S., Kutsarova, E., Ruangkittisakul, A., Zhou, H., et al. (2014). A Long Stokes Shift Red Fluorescent Ca^2+^ Indicator Protein for Two-Photon and Ratiometric Imaging. *Nat Commun* 5. doi:10.1038/ncomms6262.

Wu, J., Liu, L., Matsuda, T., Zhao, Y., Rebane, A., Drobizhev, M., et al. (2013). Improved Orange and Red Ca^2+^ Indicators and Photophysical Considerations for Optogenetic Applications. *ACS Chem Neurosci* 4, 963–972. doi:10.1021/cn400012b.

Zarowny, L., Aggarwal, A., Rutten, V. M. S., Kolb, I., Patel, R., Huang, H.-Y., et al. (2020). Bright and High-Performance Genetically Encoded Ca^2+^ Indicator Based on mNeonGreen Fluorescent Protein. *ACS Sens.* 5, 1959–1968. doi:10.1021/acssensors.0c00279.

Zhang, Y., Rózsa, M., Liang, Y., Bushey, D., Wei, Z., Zheng, J., et al. (2021). Fast and sensitive GCaMP calcium indicators for imaging neural populations. doi:10.1101/2021.11.08.467793.

Zhao, M., Hollingworth, S., and Baylor, S. M. (1996). Properties of Tri- And Tetracarboxylate Ca^2+^ Indicators in Frog Skeletal Muscle Fibers. *Biophys. J.* 70, 896–916. doi:10.1016/S0006-3495(96)79633-9.

Zhao, Y., Araki, S., Wu, J., Teramoto, T., Chang, Y.-F., Nakano, M., et al. (2011). An Expanded Palette of Genetically Encoded Ca^2+^ Indicators. *Science* 333, 1888–1891. doi:10.1126/science.1208592.

1. ∆*R*/*R*_0_ for ratiometric-pericam, GEM-GECO1, GEX-GECO1, and REX-GECO1 [↑](#footnote-ref-1)
2. ∆*R*/*R*_0_ for r-pHluorin [↑](#footnote-ref-2)
3. ∆*R*/*R*_0_ for Clomeleon and SuperClomeleon [↑](#footnote-ref-3)
